# Identification of prognostic genes associated with mitochondria and macrophage polarization in prostate adenocarcinoma based on transcriptome and Mendelian randomization analysis

**DOI:** 10.1007/s12672-025-03858-5

**Published:** 2025-12-31

**Authors:** Li Heng, Hao Bian, Chengjun Zhao, Zhen Wei, Jiancheng Cao, Guanfeng Wang

**Affiliations:** 1 Department of Urology, 3201 Hospital, HanZhong, Shaanxi China; 2 Department of Urology, Kailuan General Hospital, Tangshan, Hebei China; 3 Department of Ophthalmology, Kailuan General Hospital, Tangshan, Hebei China

**Keywords:** Prostate adenocarcinoma, Macrophage polarization, Mitochondria, Mendelian randomization, Prognostic genes

## Abstract

**Background:**

Prostate adenocarcinoma (PRAD) is a common malignancy in the male genitourinary system, with growing evidence linking its progression to mitochondrial function and macrophage polarization. This study identifies prognostic genes associated with these factors in PRAD through integrated transcriptomic data analysis and Mendelian randomization (MR).

**Methods:**

This study utilized transcriptome datasets from The Cancer Genome Atlas prostate adenocarcinoma (TCGA-PRAD). Candidate genes were selected by integrating mitochondrial-related genes (MRGs), macrophage polarization-related genes (MPRGs), and differentially expressed genes (DEGs). Prognostic genes were subsequently identified through MR and regression analyses, enabling the construction and validation of a risk prediction model. The model underwent independent prognostic assessment and nomogram validation, followed by comprehensive analyses including functional enrichment, immune profiling, and drug sensitivity evaluation comparing high- and low-risk cohorts.

**Results:**

From the overlap of 6,734 DEGs, 5,940 key module genes, and 1,136 MRGs, 103 CGs were identified. MR and regression analyses revealed seven prognostic genes (ABHD11, PTRH2, CAT, NTHL1, SLC25A39, OXR1, GSTZ1), which formed a robust risk prediction model. The model confirmed risk score, prostate-specific antigen, and Gleason score as independent prognostic factors for PRAD. A validated nomogram demonstrated high accuracy in outcome prediction. Functional enrichment analysis highlighted differential E2F target activity between risk groups, while immune profiling identified nine distinct cell populations, including immature dendritic cells. Finally, drug sensitivity analysis showed elevated IC50 values for bexarotene and CCT018159 in high-risk patients.

**Conclusion:**

This study identified seven prognostic genes and provided a new theoretical basis for exploring immune defense mechanisms and targeted therapeutic drugs in PRAD.

**Supplementary Information:**

The online version contains supplementary material available at 10.1007/s12672-025-03858-5.

## Introduction

Prostate adenocarcinoma (PRAD) is a common malignant tumor in the urinary system of middle-aged and elderly men, ranking second among global malignant tumors [1]. As of 2022, the global mortality rate of PRAD has reached 45/10,000, and in China, it has reached 15.56/100,000 [2]. PRAD patients are often asymptomatic in the early stages, with the majority being diagnosed at advanced stages characterized by bone metastasis [3]. In recent years, the global incidence of PRAD has continued to rise, primarily attributed to improved early-stage detection rates resulting from the widespread implementation of prostate-specific antigen screening [4]. However, even after standard androgen deprivation therapy, some patients still progress to metastatic castration-resistant prostate cancer [5]. Although novel approaches such as androgen receptor signaling inhibitors and immunotherapy have been employed to improve prognosis, therapeutic outcomes remain suboptimal, contributing to persistently high mortality rates [6]. Therefore, elucidating the molecular mechanisms underlying PRAD and identifying relevant biomarkers are critical for achieving early diagnosis and optimizing clinical management strategies.

Within the PRAD microenvironment, non-tumor stromal cells contribute to immune evasion and immunosuppression [7, 8]. Tumor-associated macrophages (TAMs), a dominant stromal component, promote tumor progression through angiogenesis, anti-apoptotic cytokine secretion, and pro-growth signaling [9].TAMs predominantly exhibit an M2-like phenotype associated with immunosuppression and tumor advancement [10]. Targeting M2 macrophage-derived downstream signals or blocking their tumor-promoting secretory factors may thus represent a therapeutic strategy to inhibit PRAD progression.

The mitochondrial network, functioning as the primary subcellular organelle orchestrating bioenergetic flux, metabolic homeostasis maintenance, and signal transduction modulation, intrinsically generates oxidative stress through electron transport chain-derived reactive oxygen species (ROS) biosynthesis, thereby modulating the phenotypic plasticity of macrophages towards either tumor-suppressive immunoregulation or pro-tumorigenic phenotypic shifts through redox-sensitive transcriptional reprogramming mechanisms [11]. For instance, CD11b agonist-induced mitochondrial oxidative stress in macrophages triggers endogenous mitochondrial DNA oxidation and cytoplasmic release, activating the cGAS-STING-IFN axis to drive macrophages toward anti-tumor polarization [12]. In tumor-associated macrophages, upregulated CD36 increases lipid uptake, enhancing fatty acid oxidation and mitochondrial ROS production, which activates JAK1 phosphorylation, SHP1 dephosphorylation, and STAT signaling, promoting pro-tumor TAM polarization [13]. However, there are currently no studies on how mitochondria and macrophage polarization jointly exert their biological effects and influence patient prognosis during the occurrence and development of PRAD.

Mendelian randomization (MR) analysis uses genetic variants, such as single-nucleotide polymorphisms (SNPs), that are strongly linked to exposure factors to serve as instrumental variables (IVs). This approach helps infer causal relationships between exposures and disease outcomes [14, 15]. This methodology integrates fundamental genetic principles (including Mendel’s laws of segregation and independent assortment), statistical genetics, causal inference frameworks, and disease biological mechanisms, effectively mitigating confounding biases and reverse causation while demonstrating methodological robustness. The IVs must satisfy three core assumptions: (1) Relevance assumption (strong SNP-exposure association); (2) Independence assumption (SNP independence from confounders); (3) Exclusion restriction assumption (SNPs influence outcomes solely through exposures) [16]. MR analysis has been widely applied in the etiological study of PRAD. For example, Xu et al. found through MR analysis that a short leukocyte telomere length would increase the risk of biochemical recurrence of PRAD, and the risk of Gleason score ≥ 8 in patients with short telomeres was 2.74 times that of patients with long telomeres [17, 18]. Lv et al. confirmed through MR analysis that for each one standard deviation increase in circulating phosphorus concentration, the risk of developing PRAD would increase by 19%, and there was a causal relationship between the two [19]. Lu et al. confirmed through MR analysis that for each increase of one standard deviation in serum zinc level, the risk of PRAD increases by 5.8% [20]. These studies primarily focus on epidemiological factors (e.g., telomere length, metabolites) rather than causal effects at the genetic level. Notably, MR studies integrating transcriptomic data to investigate genes regulating mitochondrial function and immune microenvironment features—key drivers of PRAD progression—remain scarce. In this study, we expand the application of MR in PRAD research by integrating transcriptomic analyses of mitochondria-related genes (MRGs) and macrophage polarization-related genes (MPRGs). Unlike previous MR studies focusing on single risk factors (e.g., telomere length, metabolites), our approach utilizes MR to validate causal relationships between candidate genes and PRAD prognosis, thereby identifying robust prognostic biomarkers and constructing a risk model. This integration addresses a critical research gap by linking genetic causality to the functional pathways of the mitochondria-macrophage axis.

This study seeks to identify prognostic genes associated with mitochondria and macrophage polarization in PRAD by integrating transcriptome data with MR analysis, thereby offering novel therapeutic targets for the disease. Based on these prognostic genes, a predictive model is developed to examine the biological pathways involved in high- and low-risk patient groups, and to investigate differences in their immune microenvironment, immunotherapy response, and drug sensitivity. This offers a new theoretical basis for researching and treating PRAD.

## Materials and methods

### Data collection

The Cancer Genome Atlas-prostate adenocarcinoma-PRAD(TCGA-PRAD) was acquired from the University of California, Santa Cruz Xena (https://xenabrowser.net/datapages/) in training set on 23th September 2024, encompassing 499 PRAD tumor tissue samples and 52 control prostate tissue samples (containing 395 samples with survival information, the number of recurrent samples was 27, and the number of non-recurrent samples was 368). The expression datasets GSE70769, which included 92 PRAD samples, among which there are 45 recurrent samples and 47 non-recurrent samples, were obtained from the Gene Expression Omnibus (GEO) (https://www.ncbi.nlm.nih.gov/geo/). GSE70769 microarray data from the Illumina platform GPL10558, including RNA sequencing data, corresponding survival information, and survival status, are used for the verification of the prognostic model. Furthermore, an aggregate of 1,136 mitochondria-related genes (MRGs) was attained from MitoCarta 3.0 (https://personal.brPRADdinstitute.org/scalvo/MitoCarta3.0/) [21](Supplementary Table 12). Then, a total of 35 macrophage polarization-related genes (MPRGs) were attained from MSigDB (https://www.gsea-msigdb.org/gsea/msigdb/). The Genome-Wide Association Studies (GWAS) data of expression Quantitative Trait Loci (eQTL) of candidate genes (CGs) were searched from the Integrative Epidemiology Unit (IEU) open GWAS (https://gwas.mrcieu.ac.uk/). The ‘’prostate cancer’’ was a keyword from the IEU open GWAS database to obtain the ukb-b-13,348 dataset. There were 9,851,867 SNPs from 462,933 samples (case: 3,269, control: 459,664). The population was European.

The TCGA-PRAD dataset consists of RNA sequencing data, stored in TSV or CSV format as a gene expression matrix. Rows represent genes with gene identifiers, columns correspond to TCGA-normalized sample IDs, and cell values are gene expression levels such as TPM/FPKM or raw counts.

The GSE70769 dataset is microarray sequencing data, formatted as a standard Series Matrix File. Rows are probe IDs from the GPL10558 platform, columns correspond to GSM sample IDs, and values are probe signal values subjected to background correction, normalization, and logarithmic transformation. The header contains metadata such as platform information and sample annotations.

### Differentially expressed genes (DEGs) analysis

In TCGA-PRAD, differential expression analysis was performed using the DEseq2 package (v 1.38.0) [22] with thresholds |log2Fold Change| >0.5 and *p* < 0.05 to identify DEGs. Top 10 up/down-regulated DEGs (sorted by log2FC) were visualized via ggplot2 package (v 3.5.1) [23], and a heatmap of 20 key DEGs was generated using the ComplexHeatmap package (v 2.14.0) [24].

### Weighted gene co-expression network analysis (WGCNA)

In TCGA-PRAD, the ssGSEA algorithm of the GSVA package (v 1.50.0) [25] was employed to evaluate the MPRGs scores of all samples, and the score disparities in MPRGs between the PRAD cohort and the control cohort were then compared (*p* < 0.05). To investigate the association between MPRGs and the prognosis of PRAD patients, patients with biochemical recurrence-free survival (BCR-FS) information were sorted into high and low MPRGs scoring cohorts by the best cutoff value of MPRGs scores. The Survminer package (v 0.4.9) [26] was applied to plot the Kaplan-Meier (KM) survival curves for patients in the two cohorts, with the log-rank test used to determine the difference in survival between them (*p* < 0.05).WGCNA (v 1.72) [27] was used to identify modules associated with MPRGs. After outlier removal via GoodSamplesGenes, a soft threshold was selected based on scale-free topology. Gene modules were constructed using hierarchical clustering (minimum 100 genes/module, merge cut height = 0.25), and key modules were defined by correlation with phenotypic traits (|cor| >0.3, *p* < 0.05).

### Screening and enrichment analysis of CGs

The VennDiagram package (version 1.7.3) was utilized to determine the intersection of DEGs, key module genes, and MRGs, then CGs were obtained [28]. To delve deeper into the biological processes underlying the CGs, Gene Ontology (GO) analysis and KEGG annotation were executed using the clusterProfiler package (v 4.10.1) (*p* < 0.05) [29].

### Mendelian randomization(MR) analysis

CGs were analyzed as exposure factors influencing PRAD risk using a two-sample MR approach. IVs were selected based on genome-wide significant SNPs (*p* < 5 × 10⁻⁷) strongly associated with CGs, adhering to three core MR assumptions: (1) relevance (SNP-exposure association), (2) independence (no confounding bias), and (3) exclusion restriction (SNPs affect PRAD solely via CGs). Linkage disequilibrium (LD) pruning (r² < 0.001, kb = 10) was applied to ensure SNP independence using the VariantAnnotation and ieugwasr packages [30, 31]. Allele harmonization and weak IV exclusion (F-statistic < 10) were performed via the TwoSampleMR package [32]. Causal effects were estimated using five MR methods: inverse-variance weighted (IVW, primary method), MR-Egger, weighted median, simple mode, and weighted mode. Among them, the IVW method served as the primary measure for determining statistical significance (*p* < 0.05) [32–36]. Effect directions were interpreted as risk (odds ratio (OR) >1) or protective (OR < 1), visualized through scatterplots, forest plots, and funnel plots. Sensitivity analyses included: Heterogeneity: (1) Cochran’s Q test (*p* >0.05) [37]; (2) Horizontal pleiotropy: MR-Egger intercept test (*p* >0.05) and MR-PRESSO (NbDistribution = 10,000, Outliers were left empty) [14]; (3) Robustness: Leave-one-out analysis to confirm stability of causal estimates [38]. (4) Directionality: Steiger test validating unidirectional causality (*p* < 0.05) [39].

CGs with concordance between MR-derived ORs and differential expression trends in PRAD were prioritized for prognostic model construction. This multi-layered validation framework reduced confounding and reverse causation, ensuring robust inference of causal gene-PRAD relationships. eQTLs underwent quality control (weak-effect variants excluded), followed by Effective-Median-based Mendelian Randomization (EMIC) analysis. Using PLINK, LD matrices were computed for each MR-derived gene to estimate causal effect correlation in the EMIC model (negative effect size indicated potential decreased outcome risk with higher exposure). Confidence intervals (e.g., 95% CI = effect size ± 1.96 × standard error) were calculated via standard error to define plausible effect size ranges; exposure-outcome association was statistically significant at *p* < 0.05, supporting a causal relationship.

The genomic control inflation factor (λGC) tested genetic background similarity across datasets. Points above the diagonal indicated severe p-value inflation; those on the diagonal showed observed -log10(P) matched theoretical expectations, fitting the no-association null hypothesis (λGC = 1, ideal state) with no population stratification, technical bias, or significant genetic associations, serving as an ideal GWAS benchmark. Points below the diagonal meant observed -log10(P) was smaller than expected (Pobs > P_exp), possibly due to insufficient statistical power (e.g., small samples unable to detect weak associations).

### Construction of risk models

In TCGA-PRAD, the survival package (v 3.5.3) was employed to execute univariate Cox analysis on CGs (hazard ratio (HR) ≠ 1, *p* < 0.2) [40]. Following the findings from the univariate analysis, the PH assumption was tested. Subsequently, the forestplot package (v 2.0.1) was applied to display the results of candidate prognostic genes [41]. Glmnet package (v 4.1.1) was then used to conduct least absolute shrinkage and selection operator (LASSO) regression analysis on candidate prognostic genes (with 10-fold cross-validation) [41]. Moreover, prognostic genes were identified when lambda reached lambda.min. Thereafter, in TCGA-PRAD, risk scores for each PRAD sample were calculated by the relative expression intensity of prognostic genes and the coefficients obtained from LASSO regression. The risk scores were calculated as the following formula: $\:\text{R}\text{i}\text{s}\text{k}\text{s}\text{c}\text{o}\text{r}\text{e}\:=\:\sum\:_{\text{i}\:=\:1}^{\text{n}}{\upbeta\:}\left({\text{g}\text{e}\text{n}\text{e}}_{\text{i}}\right)\text{*}\text{x}\left({\text{g}\text{e}\text{n}\text{e}}_{\text{i}}\right)$, where x represents the sample expression levels corresponding to the gene, and $\:{\upbeta\:}$ represents the linear regression coefficient. TCGA-PRAD was utilized as a training dataset (*n* = 394) (samples with less than 30 days of recurrence were removed) to construct a risk model, while GSE70769 dataset served as a validation dataset (*n* = 90) (samples with less than 30 days of recurrence were removed) to validate risk model for predicting PRAD sample outcomes.We set the minimum sample size of the GSE70769 validation set to account for at least 40% of the total sample size, aiming to address the potential imbalance in sample size between different risk groups.

Subsequently, a scatter plot of risk curves was generated to depict the spread of risk assessments and the survival outcomes for patients with PRAD using the ggrisk package (v 1.3) (https://rdocumentation.org/packages/ggrisk/versions/1.3). Additionally, the prognostic gene expression profiles were illustrated, and a KM survival plot was constructed to assess the disparity in survival rates between two distinct risk cohorts, with the survival package (v 3.5.3) (*p* < 0.05) [40]. The receiver operating characteristic (ROC) curve, which calculates the area under the curve (AUC) for 1, 3, and 5 years, was plotted using the survivalROC package (v 1.0.3) to evaluate the prognostic model’s effectiveness, with an AUC >0.6 indicating good predictive performance [40]. Concurrently, a heatmap was applied to depict the expression of prognostic genes across two risk cohorts with the pheatmap package (v 1.0.12) [24]. Furthermore, the risk model was validated in an independent validation dataset.

### Independent prognostic analysis

In TCGA-PRAD (with information on BCR-FS), the risk score and clinical characteristics (age, prostate-specific antigen, Gleason, N.stage) were included in both regression analyses to obtain independent prognostic factors (HR ≠ 1, *p* < 0.05). To evaluate the prognostic significance of the independent risk factors for PRAD, the rms package (v 6.5-0) was applied to build a nomogram by the independent prognostic factors [24]. Predictive model based on independent prognostic factors, rms package (v 6.5-0) was applied to build calibration curves for 1, 3, and 5 years for the nomogram (the accuracy of the model’s predictions was indicated by how close the slope of the calibration curve was to 1). TimeROC package (v 0.4) was applied to plot the ROC curves for the nomogram of the independent prognostic factors, to validate the nomogram’s predictive capabilities (AUC >0.6) [42].

### Enrichment analysis

To evaluate the biological pathways correlated with prognostic genes in PRAD (with information on BCR-FS), the DEGs (HRC vs. LRC) were analyzed using the DESeq2 package (v 1.38.0), which were sorted according to log2FC in ascending order. Moreover, gene set enrichment analysis (GSEA) (*p* < 0.05, |normalized enrichment score (NES)| >1) was carried out via the clusterProfiler package (v 4.10.1), with “h.all.v2023.2.Hs.symbols.gmt” from the MSigDB database (https://www.gsea-msigdb.org/gsea/msigdb/) as reference gene set. The GOplot package (v 1.0.2) was employed to show the top 5 pathways in descending order (*p* < 0.05) [43]. The GSVA package (v 1.50.0) was employed to evaluate pathway scores related to samples in the HRC and LRC. Furthermore, the disparities in HALLMARK pathway scores between HRC and LRC were compared (*p* < 0.05, |t| >2.0), and a bar chart was drawn to display the significantly different HALLMARK (t >0, the pathway was considered activated in HRC, t < 0, the pathway was considered inhibited in HRC).

### DNA damage repair analysis

In TCGA-PRAD (with information on BCR-FS), patients were sorted into HRC and LRC by their risk status. DNA damage repair pathways were used as the reference gene set. The GSVA package (v 1.50.0) was employed to assess the pathway scores for samples in both HRC and LRC. Then the variance in pathway scores between HRC and LRC was compared (*p* < 0.05, |t| >2.0). Finally, the ggplot2 package (v 3.5.1) was utilized to visualize the results.

### Immune-related analyses

In TCGA-PRAD (with BCR-FS information), immune cell infiltration (28 types) was evaluated using the ssGSEA algorithm via the GSVA package (v1.50.0) [44]. We chose ssGSEA over other deconvolution methods because it quantifies individual sample gene set activity through non-parametric ranking and ECDF difference calculation, without relying on group information—suitable for single-cell/small sample heterogeneity analysis. Its non-parametric nature reduces sample size-related bias, complementing GSVA (distribution-dependent) and traditional GSEA (group-ranking dependent); it is also efficient and supports custom gene sets/parameter adjustment [25].Results were visualized using the pheatmap package (v1.0.12). Differences in immune cell infiltration between HRC and LRC were compared (*p* < 0.05), with immune scores visualized via the ggplot2 package (v3.5.1) (*p* < 0.05). Spearman’s analysis (psych package, v2.4.3) determined correlations between prognostic genes/differential immune cells and between risk scores/differential immune cells (|cor| >0.3, *p* < 0.05) [45]. In TCGA-PRAD, immune checkpoint gene expression differences between HRC and LRC were compared (*p* < 0.05), using common checkpoints from previous studies [45]. Distribution differences of HRC/LRC across immune subtypes were analyzed (*p* < 0.05); Wilcoxon and Kruskal-Wallis tests assessed variations in risk scores and prognostic gene expression across subtypes (*p* < 0.05). To evaluate immunotherapy response, Tumor Immune Dysfunction and Exclusion (TIDE) and Tumor Mutation Burden (TMB) scores were calculated via the TIDE algorithm (http://tide.dfci.harvard.edu/login/). IPS was computed using The Cancer Imaging Archive (https://tcia.at/) for patients under different treatments, with IPS differences between HRC and LRC analyzed (*p* < 0.05).

### Drug sensitivity analysis

To evaluate the differential response to chemotherapy between HRC and LRC patient groups, the Genomics of Drug Sensitivity in Cancer (GDSC) database (http://www.cancerrxgene.org/) was used to identify common chemotherapeutic agents. The oncoPredict package (v 0.2) was employed to calculate the 50% inhibiting concentration (IC50) values for common chemotherapy and molecular targeted drugs in all samples [46]. Subsequently, the IC50 differences were compared between HRC and LRC (*p* < 0.05), and the top 5 drugs with the most notable differences were selected for presentation (p.adj < 0.05).

### The validation of the prognostic genes

To verify the consistency of prognostic gene expression in clinical samples with the bioinformatics results. The Wilcoxon test was applied to compare the differences in prognostic gene expression in the PRAD cohort and control cohort (*p* < 0.05). To further validate the expression patterns of the identified prognostic genes in vitro, we analyzed their expression levels across a panel of PRAD cell lines with distinct molecular characteristics. PRAD cell lines (VCaP, LNCaP, C4-2, LNCaP-95, LNCaP-abl, 22RV1, PC3, DU145, NCI-H660) were obtained from previous studies [47].

### Statistical analysis

Data were analyzed using R software (version 4.2.2) and GraphPad Prism version 8.0. The Wilcoxon rank-sum test was employed for comparisons, with statistical significance set at *p* < 0.05.

## Results

### Obtaining DEG and essential module genes

**Fig. 1:**
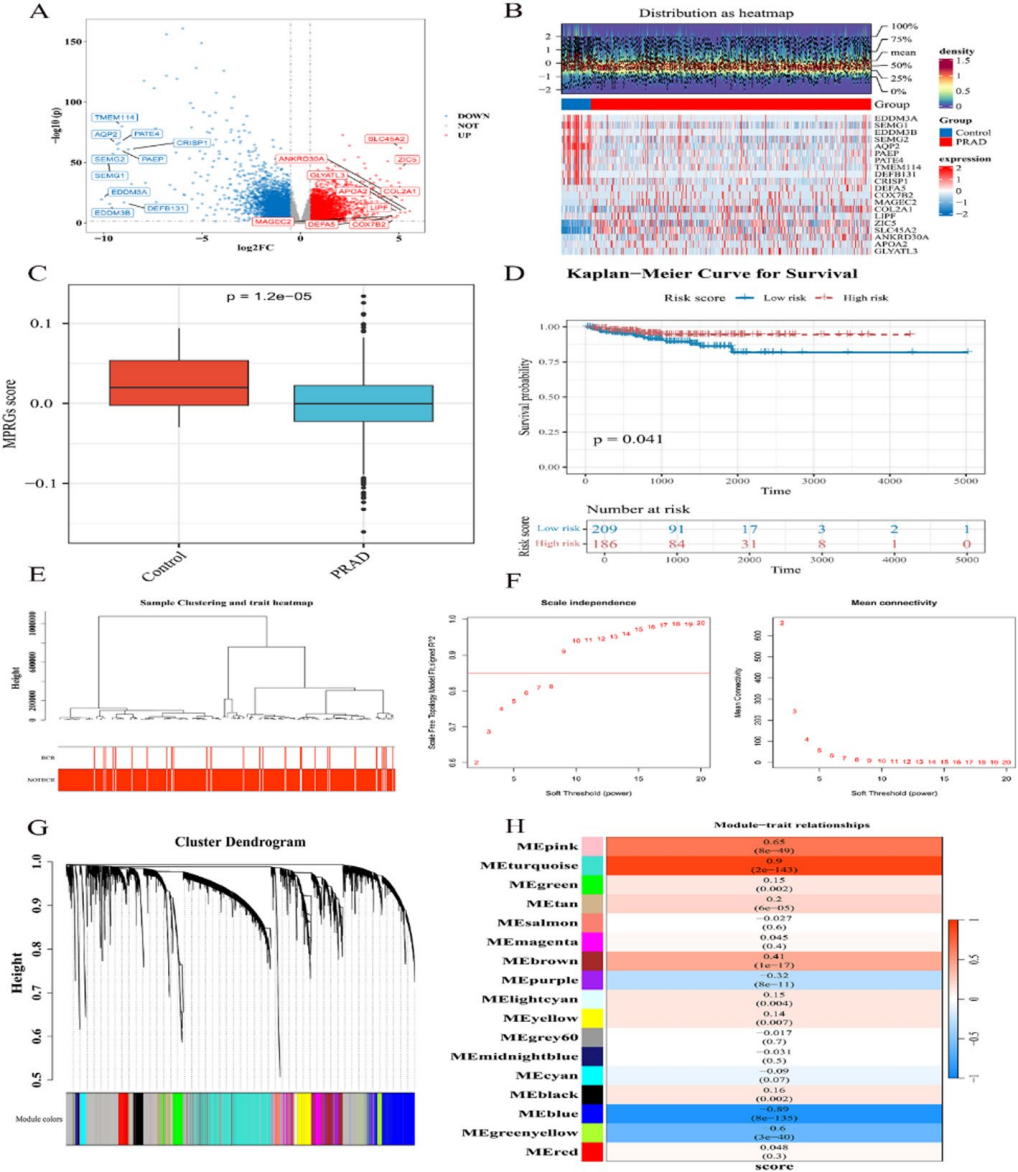
Acquisition of differentially expressed genes (DEGs) (**A**). Volcano plot of DEGs. The red points represent upregulated genes, the blue points represent downregulated genes, and the gray points represent genes with no significant expression. The gene names of the top 10 genes with the most significant upregulation and downregulation DEGs. **B** Heatmap of DEGs between the prostate adenocarcinoma (PRAD) and control groups. In this figure, the top 10 downregulated genes and the top 10 upregulated genes, sorted by the fold change log2FC, are plotted. **C** Box plot of ssGSEA score. between the PRAD and control groups. **D** The Kaplan-Meier (KM) survival curve of high and low score groups of macrophage polarization-related genes (MPRGs) in the validation datasets. **E** Hierarchical clustering analysis tree. Each branch in the clustering tree represents a sample. The red color blocks at the bottom represent the BCR group and the NOTBCR group, respectively. **F** Selection of soft threshold. The horizontal axes of both the left and right graphs represent the weight parameter power value. The vertical axis of the left graph represents the evaluation coefficient of the scale-free network. The vertical axis of the right graph represents the average connectivity. **G** The clustering dendrogram of transcriptomics, each leaf corresponds to a different gene module. **H** Heatmap of correlations between modules and traits, each cell containing the corresponding correlation and p-value

In TCGA-PRAD, 6,734 DEGs (3,259 upregulated, 3,475 downregulated) were identified between PRAD and control groups (Fig. 1A-B). MPRG scores were higher in controls than PRAD (*p* < 0.05), and high MPRG scores correlated with poorer BCR-FS (*p* = 0.041, Fig. 1C-D). WGCNA revealed no outliers and identified a soft threshold (power = 9) for scale-free network construction (Fig. 1E-F). 16 modules were clustered, with MEturquoise (3,893 genes, cor = 0.9, *p* = 2 × 10^–143^) and MEblue (2,047 genes, cor = -0.89, *p* = 8 × 10^–135^) as key modules (|cor| >0.3, *p* < 0.05), yielding 5,940 key module genes (Fig. 1G-H).

### Identifying candidate genes (CGs) and crucial biological pathway analysis

**Fig. 2:**
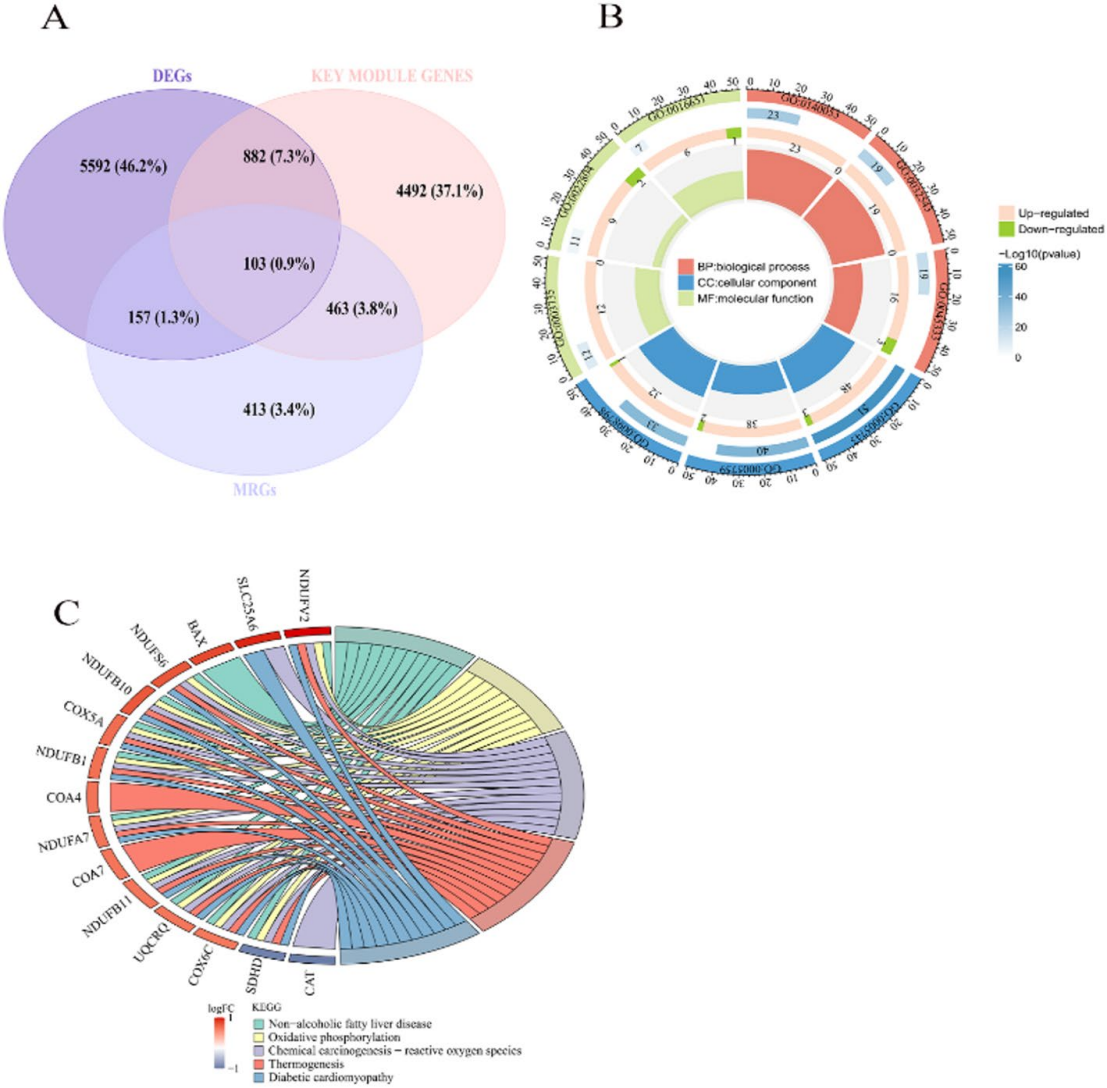
Identifying and analyzing candidate genes for functional enrichment. **A** Venn diagram of the shared genes between the DEGs, key module genes, and MRGs. **B** Significantly enriched GO terms for candidate genes (CGs). **C** Kyoto Encyclopedia of Genes and Genomes (KEGG) enrichment analysis of CGs

A sum of 103 CGs was obtained by taking the intersection of 6,734 DEGs, 5,940 key module genes, and 1,136 MRGs (Fig. 2A). A total of 309 entries were obtained in GO enrichment, of which 209 were enriched for biological process (BP), 44 for cellular component (CC), and 56 for molecular function (MF), such as structural constituent of ribosome, mitochondrial translation, and mitochondrial matrix (Supplementary Table 1, Fig. 2B). Additionally, 111 KEGG pathways were discovered, including non-alcoholic fatty liver disease and thermogenesis. (Supplementary Table 2, Fig. 2C).

### MR analysis

A two-sample MR analysis was conducted with 103 CGs as exposure factors and PRAD as the outcome. Through IVW, 13 genes were identified (*p* < 0.05), including 11 risk factors (C15orf61, PTRH2, TIMM13, COA4, COA7, SLC25A39, HSPE1, GSTZ1, ABHD11, COMTD1, NTHL1) with OR > 1 and 2 protective factors (OXR1, CAT) with OR < 1 (Supplementary Table 3). Scatter plots showed positive correlations for the 11 risk factors and negative correlations for OXR1 and CAT (Supplementary Fig. 1), while forest plots confirmed effect values > 0 for risk factors and < 0 for protective factors (Supplementary Fig. 2). Additionally, SNP numbers were largely symmetrical around the IVW line, conforming to Mendel’s second law (Supplementary Fig. 3).

Sensitivity analyses validated the robustness of these results: all 13 genes showed no heterogeneity (*p* > 0.05; e.g., OXR1 and CAT with I² = 0) (Supplementary Table 4), no horizontal pleiotropy (*p* > 0.05) (Supplementary Table 5), and no significant bias in LOO analysis (Supplementary Fig. 4). The Steiger test ruled out reverse causality, with a “TRUE” directional relationship indicating no evidence that the 13 genes are affected by PRAD (*p* < 0.05) (Supplementary Table 6).

Further analysis of these 13 genes via the EMIC model (exemplified by OXR1) retained 31 SNPs (R² ≥ 0.2), revealing a weak but significant causal relationship (effect size = -0.00185, *p* = 0.00345) (Supplementary Table 13). λGC evaluation showed severe p-value inflation for OXR1 (Supplementary Fig. 5).

### Recognition of seven prognostic genes

**Fig. 3:**
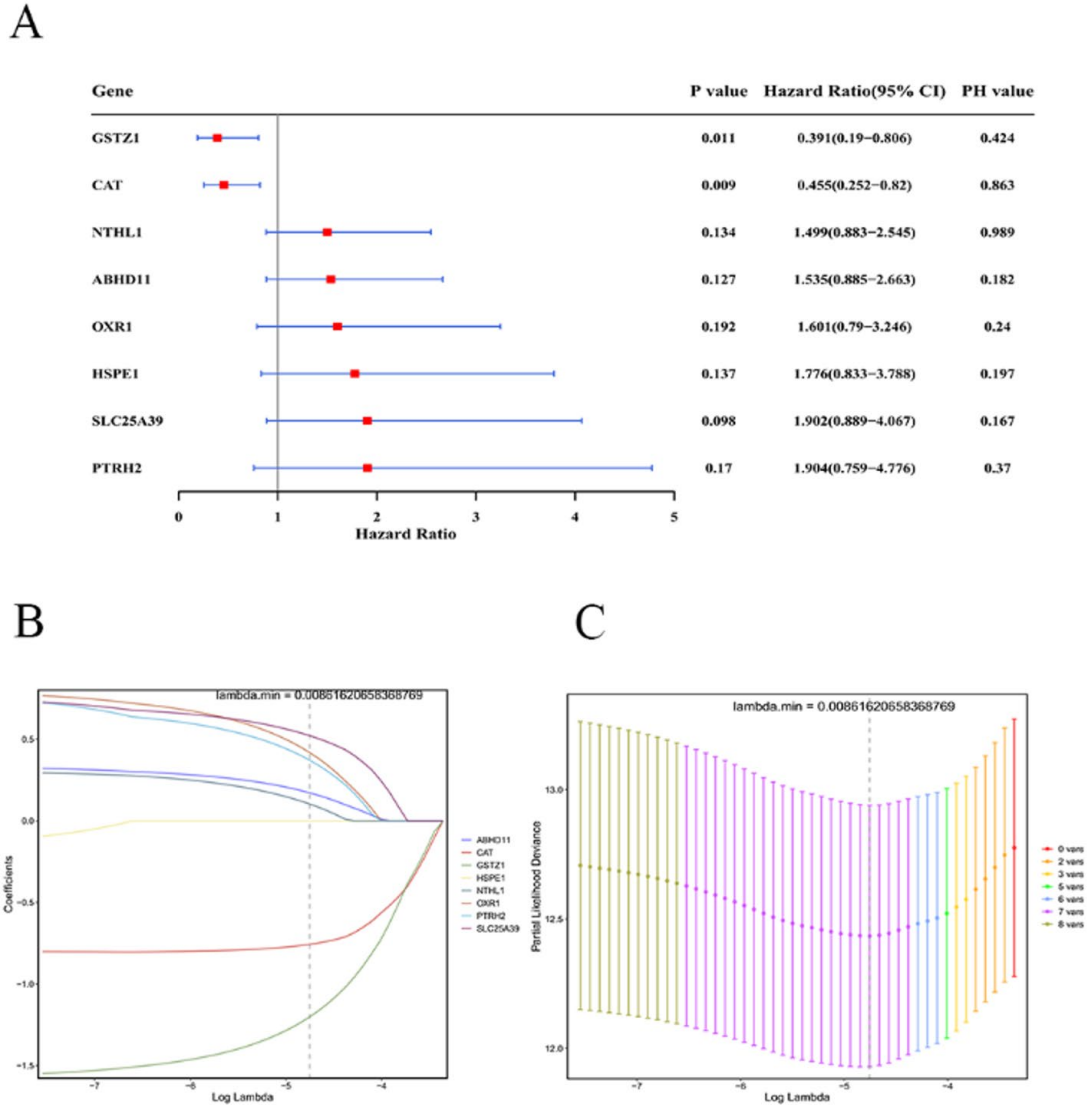
Construction of a risk model based on the candidate prognostic genes in PRAD. **A** Univariate Cox regression analysis of prognosis-related genes. Genes with a hazard ratio (HR) > 1 are risk genes, and genes with an HR < 1 are protective genes. **B**, **C** Least absolute shrinkage and selection operator (LASSO) regression

A total of 8 genes passed the proportional hazards (PH) assumption test (*p* > 0.05) and were considered as potential predictive genes, which were GSTZ1, CAT, NTHL1, ABHD11, OXR1, HSPE1, SLC25A39, and PTRH2 (Fig. 3A). Ultimately, seven prognostic genes were identified, which were PTRH2, SLC25A39, OXR1, ABHD11, NTHL1, CAT, and GSTZ1 (lambda.min = 0.00861620658368769) (Fig. 3B-C).

### Construction of a risk model with high accuracy

**Fig. 4:**
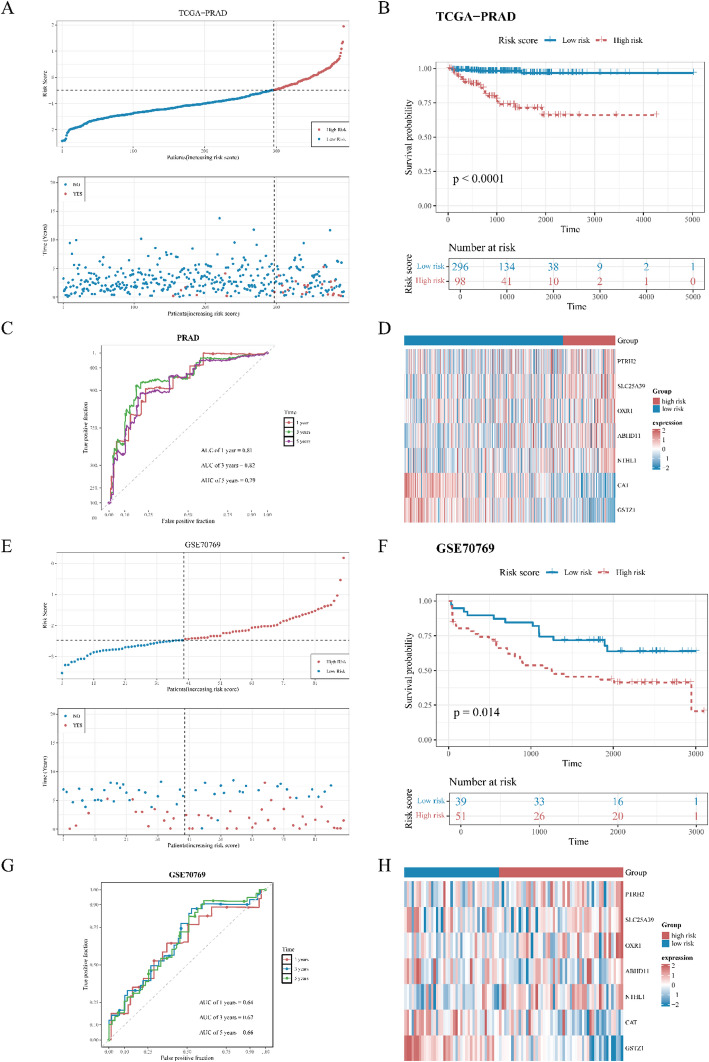
**A**-**D** Assessment of a risk model using the Cancer Genome Atlas for prostate adenocarcinoma (TCGA-PRAD). **A** The risk curve and survival status distribution of TCGA-PRAD patient samples in the training set. The upper part of the figure: High-risk patients are indicated by red dots, while low-risk patients are indicated by blue dots; The lower part of the figure: The red dots represent deceased patients, and the blue dots represent surviving patients. **B** KM curve of high and low risk groups of TCGA-PRAD in the training set. Red represents the high-risk group, and blue represents the low-risk group. **C** The receiver operating characteristic (ROC) curve of the model at 1-, 3-, and 5-year in the training set TCGA-PRAD. **D** Expression heatmap of the prognostic CGs in the testing set. (E-H) Validation of risk model based on Gene Expression Omnibus (GEO) cohorts. **E** The risk curve and survival status distribution of samples in the validation set GSE70769. The upper part of the figure: The red dots represent high-risk patients, and the blue dots represent low-risk patients. The lower part of the figure: The red dots represent deceased patients, and the blue dots represent surviving patients. **F** KM curve of high- and low-risk score groups in the GSE70769. Red represents the high-risk group, and blue represents the low-risk group. **G** The ROC curve of the model at 1-, 3-, and 5-year in the GSE70769. **H** Expression heatmap of the prognostic candidate genes (CGs) in the GSE70769

Therefore, a risk model was built by expression intensity and risk coefficients of seven prognostic genes. Constructed risk model was as followed:$$ {\text{RiskScore}}{\mkern 1mu} =$$$$ {\mkern 1mu} {\text{PTRH2 }} \times {\text{ }}0.{\text{37}}{\mkern 1mu} +$$$$ {\mkern 1mu} {\text{SLC25A39 }} \times {\text{ }}0.{\text{52}}{\mkern 1mu} + {\mkern 1mu} $$$$ {\text{OXR1 }} \times {\text{ }}0.{\text{42}}{\mkern 1mu} + $$$$ {\mkern 1mu} {\text{ABHD11 }} \times {\text{ }}0.{\text{17}}{\mkern 1mu} +$$$$ {\text{NTHL1 }} \times {\text{ }}0.{\text{1}}0{\mkern 1mu} + {\mkern 1mu} {\text{CAT }} \times {\text{ }} - $$$$ 0.{\text{76}}{\mkern 1mu} + {\mkern 1mu} {\text{GSTZ1 }} \times {\text{ }} - {\text{1}}.{\text{2}}0$$ model, PRAD patients were split into an HRC (*n* = 98) and an LRC (*n* = 296) (cut-off value = -0.493748) in TCGA-PRAD. Patients’ risk scores increased from low risk to high risk, and the number of deaths among HRC patients was notably higher than that among LRC patients (Fig. 4A). There was a notable difference in survival between the two cohorts (*p* < 0.0001), with patients in HRC having a lower survival rate (Fig. 4B). Moreover, the AUC values for 1-, 3-, and 5-year were 0.81, 0.82, and 0.79, respectively. this suggests that the model demonstrated superior predictive accuracy (AUC > 0.7) (Fig. 4C). Then, the expression of prognostic genes in the HRC and LRC was shown in Fig. 4D. On the other hand, in GSE70769, after adjusting the grouping parameter (minimum sample size of either cohort ≥ 40% of total samples), patients were divided into HRC (*n* = 51) and LRC (*n* = 39) (cut-off value =-2.474466). As patients’ risk scores escalated from the low risk to the high risk, there was a marked increase in the mortality rate within the HRC compared to the LRC (Fig. 4E). Moreover, there remained a notable difference in survival between the two cohorts (*p* < 0.05), with those in HRC exhibiting a reduced survival probability (Fig. 4F). This indicated that the significant prognostic value of the model was retained even after balancing the sample ratio. Additionally, the AUC values for 1-, 3-, and 5-year were 0.64, 0.67, and 0.66, respectively, suggesting the model possessed strong predictive capabilities (Fig. 4G). Finally, the expression of prognostic genes in the HRC and LRC was shown in Fig. 4H.

### Establishing a nomogram and crafting calibration curves to foresee the outcomes of patients with PRAD

**Fig. 5:**
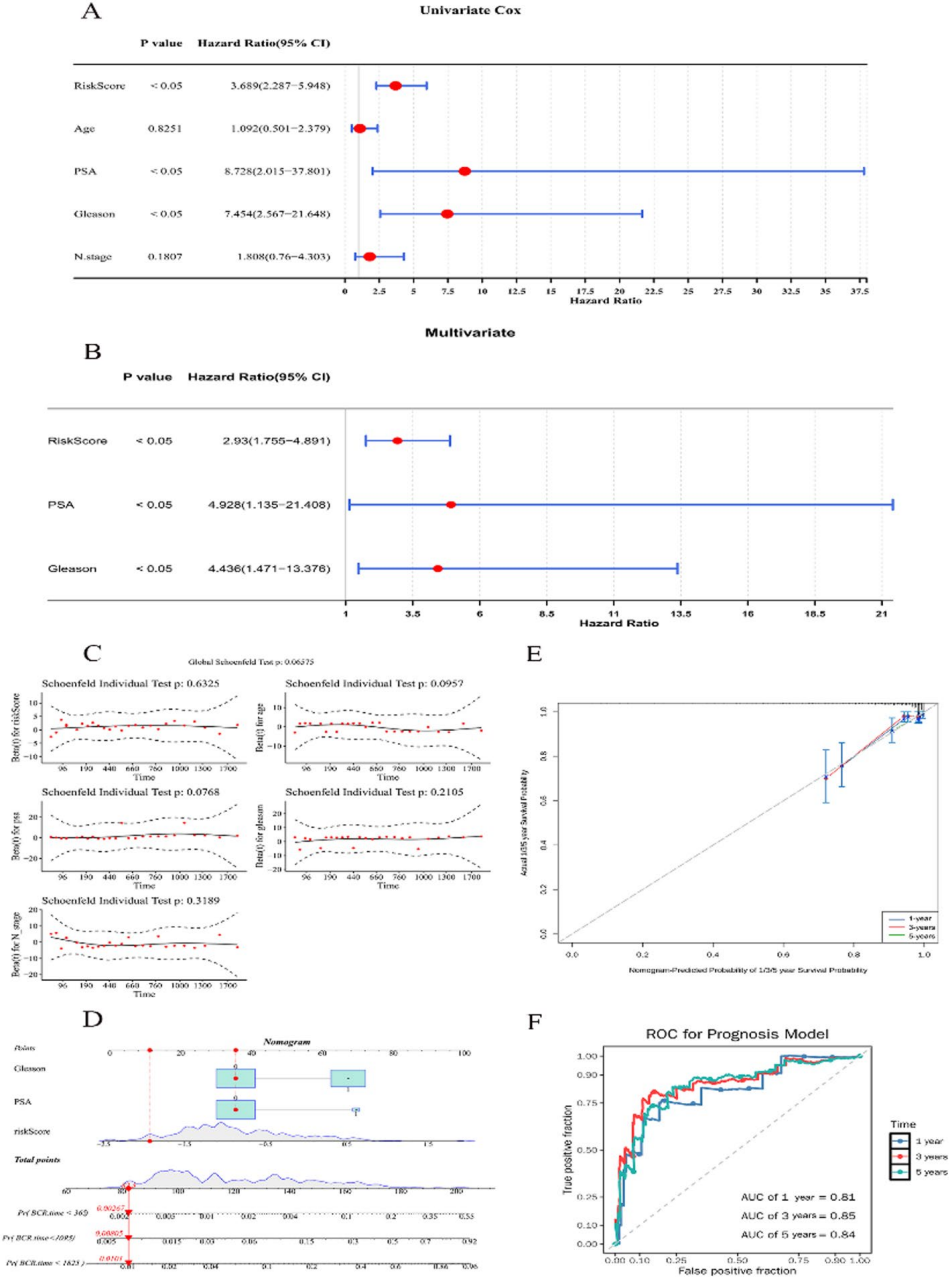
The prognostic signatures of CGs for PRAD are independent. **A** Univariate and (**B**) multivariate Cox analyses demonstrate the relationship between BCR-free occurrence, risk score, and clinicopathological factors. **C** The proportional hazards (PH) assumption test plot of univariate regression analysis. The horizontal coordinate is time, and the vertical coordinate is five clinical characteristics. **D** The nomogram model was constructed based on independent prognostic factors. **E** Calibration analysis revealed a high degree of accuracy in predicting survival at 1-, 3-, and 5-year. **F** The ROC curve of the nomogram of independent prognostic factors

Risk score, prostate-specific antigen, and Gleason were recognized as independent prognostic factors of TCGA-PRAD. 3 independent prognostic factors were risk factors (hazard ratio, HR > 1 and *p* < 0.05) (Fig. 5A-B). Moreover, all p-values were greater than 0.05 in the PH assumption test (Fig. 5C). Advancing our analysis, we integrated these independent prognostic factors into a nomogram (Fig. 5D). The slope of the calibration curve was closer to 1, which reflected the nomogram’s high predictive precision for patient outcomes at 1-, 3-, and 5-year intervals (Fig. 5E). ROC curve analysis of 1-, 3-, and 5-year in TCGA-PRAD were 0.81, 0.85, and 0.84, respectively, which suggested that the predictive performance of the nomogram plot was excellent (Fig. 5F).

### Biological pathway of risk groups in PRAD

**Fig. 6:**
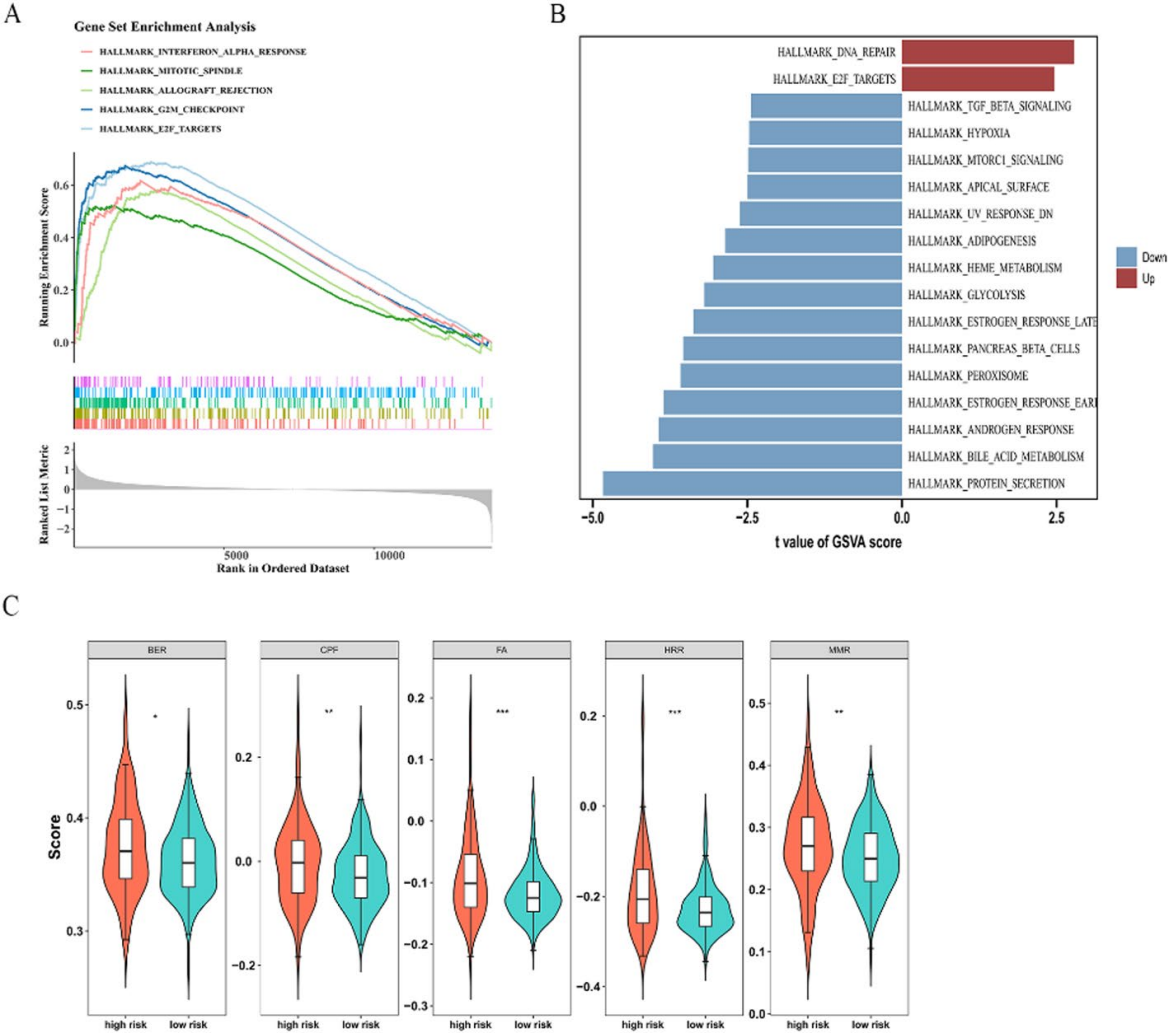
Functional enrichment analysis of the differentially expressed prognostic genes in the different risk groups. **A** GSEA enrichment analysis of differentially expressed genes between high- and low-risk groups. **B** Bar chart of significantly different HALLMARK pathways between high- and low-risk groups. Red indicates the up-regulated pathways in the high-risk group, and blue indicates the down-regulated pathways in the high-risk group. **C** Violin plot of DNA damage repair (DDR) pathways. The 5 subplots respectively represent 5 DDR pathways. The horizontal coordinate is the high and low risk groups. Red is the high-risk group, and cyan is the low-risk group. The vertical coordinate is the ssGSEA score. *BER* base excision repair, *CPF* checkpoint factors, *FA* Fanconi anemia pathway, *HRR* homologous recombination repair, and *MMR* mismatch repair pathways ***: *p* < 0.001, **: *p* < 0.01, *: *p* < 0.05

In TCGA-PRAD (BCR-FS information), the GSEA of all genes identified 25 pathways in two risk groups, like allograft rejection, G2M checkpoint, and E2F targets (*p* < 0.05) (Fig. 6A, Supplementary Table 7). Gene set variation analysis (GSVA) of all genes identified 17 pathways, DNA repair, and E2F targets were activated in HRC (t > 0). Other pathways, like protein secretion and bile acid metabolism, were restrained in HRC (t < 0) (Fig. 6B, Supplementary Table 8). The common pathway “E2F targets” had significant implications for the impact on PRAD in both GSEA and GSVA. Moreover, the base excision repair (BER), checkpoint factors (CPF), Fanconi anemia pathway (FA), homologous recombination repair (HRR), and mismatch repair (MMR) pathways showed significant differences, with all five pathways being notably up-regulated in the HRC (*p* < 0.05) (Fig. 6C).

### Analysis of immune cell infiltration and assessment of immunotherapy response in risk groups

**Fig. 7:**
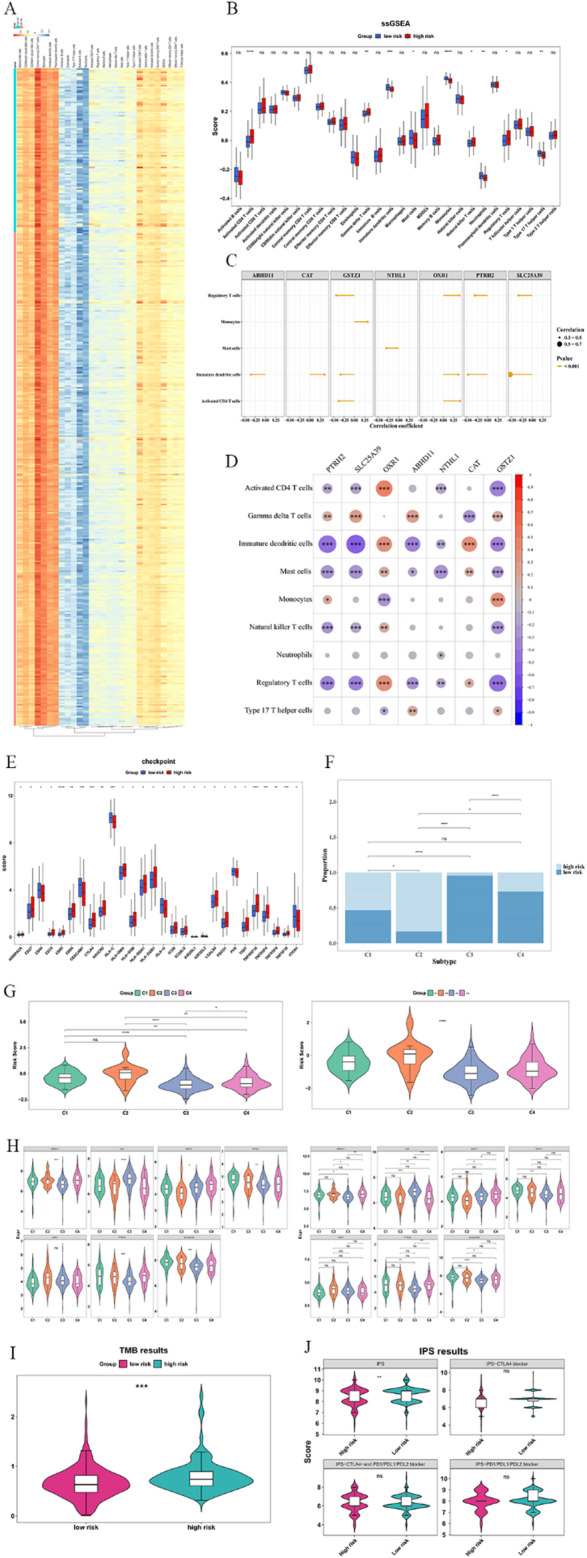
Immune infiltration analysis of PRAD patients between high- and low-risk groups. **A** Heatmap of the proportion of immune cell infiltration in the high and low risk groups. The columns are samples, and the rows are immune cells. **B** The differences in immune cells between the high- and low-risk groups of prognostic genes. **C**, **D** The correlation between prognostic genes and differential immune cells. **E** The differences in immune checkpoints between high- and low-risk groups. **F** The distribution differences of high- and low-risk groups in different immune subtypes. **G** The distribution differences of risk scores among different immune subtypes. The left figure shows the results of the Wilcoxon test, and the right figure shows the results of the Kruskal-Wallis test. The horizontal coordinate of both figures represents different immune subtypes, and the vertical coordinate is the risk score. **H** The distribution differences of prognostic genes among different immune subtypes. The left figure shows the results of the Wilcoxon test, and the right figure shows the results of the Kruskal-Wallis test. **I** The difference in TMB score between high- and low-risk groups. **J** The difference graph of the IPS score between high- and low-risk groups. *C1* wound healing type, *C2* IFN-γ dominant, *C3* inflammatory type, *C4* lymphocyte depletion) (ns *p* > 0.05, *: *p* < 0.05, **: *p* < 0.01, ***: *p* < 0.001, ****: *p* < 0.0001)

The percentage distribution of 28 immune cell infiltrates is shown in Fig. 7A. 9 cell types differed significantly between HRC and LRC: activated CD4 T cells, gamma-delta (γδ) T cells, natural killer T cells, and regulatory T cells (Tregs) were upregulated in HRC, while the remaining were upregulated in LRC (Fig. 7B).

Association analysis revealed: OXR1 strongly correlated with activated CD4 T cells (cor = 0.40, *p* < 0.001); NTHL1 with mast cells (cor=-0.30, *p* < 0.001); GSTZ1 with Tregs (cor=-0.44, *p* < 0.001); PTRH2 (cor=-0.47, *p* < 0.001), SLC25A39 (cor=-0.53, *p* < 0.001), ABHD11 (cor=-0.35, *p* < 0.001), and CAT (cor = 0.36, *p* < 0.001) with immature DCs. Risk scores showed no significant correlation with differential immune cells (|cor| < 0.3, *p* > 0.05) (Fig. 7C-D, Supplementary Table 9).

Of 79 immune checkpoints, 28 differed between HRC and LRC (e.g., CD80, TNFRSF18, TNFRSF4; Fig. 7E). Among immune subtypes, only C1 (wound healing) and C4 (lymphocyte depletion) showed no distribution differences between HRC and LRC (Fig. 7F). Risk scores differed significantly between C1 vs. C3, C2 vs. C3, C2 vs. C4, C3 vs. C4, and across all four subtypes (Fig. 7G). ABHD11, PTRH2, CAT, NTHL1, and SLC25A39 differed between C1 and C3; GSTZ1 between C2 and C3; all except OXR1 differed across the four subtypes (Fig. 7H). HRC had higher TMB scores (Fig. 7I), and IPS differed significantly between groups (Fig. 7J).

### Identifying chemotherapeutics associated with risk score in PRAD

**Fig. 8:**
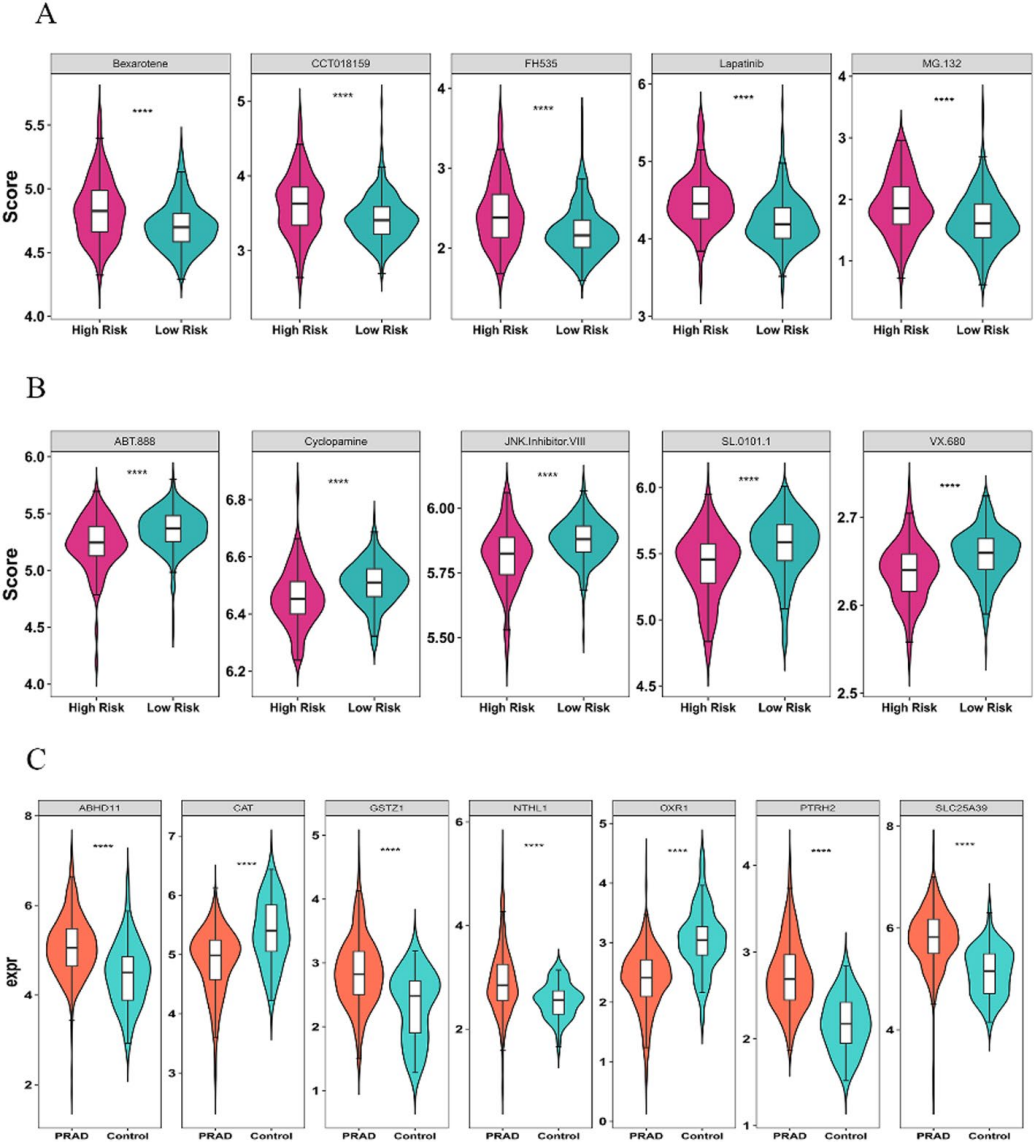
Analysis of drug sensitivity among PRAD patients across various risk categories **A** The drugs with higher IC50 values are in the high-risk group. **B** The drugs with lower IC50 values are in the high-risk group. Red represents the high-risk group, and green represents the low-risk group. **C** The expression difference diagram of prognostic genes between the PRAD group and the control group. (****: *p* < 0.0001)

A number of 24 drugs had notably higher IC50 values in the high-risk category (*p* < 0.05) (Supplementary Table 10). The top 5 drugs were presented according to their p.adj value, which were Bexarotene, CCT018159, FH535, Lapatinib, and MG.132 (p.adj < 0.05) (Fig. 8A). A number of 45 drugs had notably lower IC50 values in the high-risk category (*p* < 0.05) (Supplementary Table 11). The top 5 drugs were presented according to their p.adj value, which was ABT.888, Cyclopamine, and JNK Inhibitor, VIII, SL.0101.1, VX.680 (p.adj < 0.05) (Fig. 8B).

### Prognostic gene expression levels

**Fig. 9:**
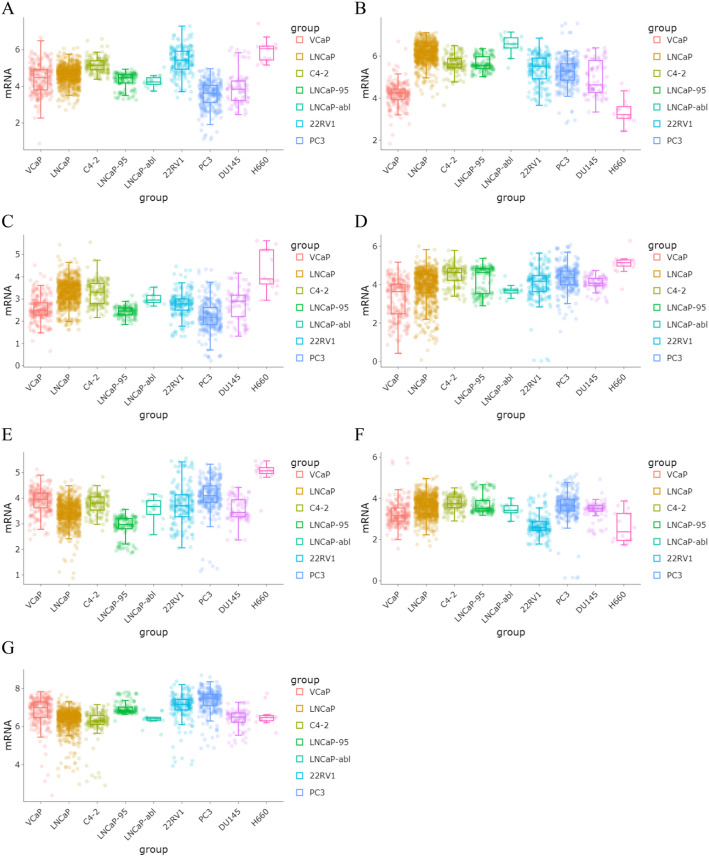
Expression levels of prognostic genes in 9 PRAD cell lines. **A** ABHD. **B** CAT. **C** GSTZ1. **D** NTHL1. **E** OXR1. **F** PTRH2. **G** SLC25A39. The vertical and horizontal axes represent the expression values of genes, while the horizontal axis represents different cell lines

In TCGA-PRAD, ABHD11, GSTZ1, NTHL1, PTRH2, SLC25A39, CAT, and OXR1 exhibited significant differences between the PRAD and control groups. The expression levels of ABHD11, GSTZ1, NTHL1, PTRH2, and SLC25A39 were notably up-regulated in PRAD samples (*p* < 0.05), Conversely, the expression of CAT and OXR1 was down-regulated in the PRAD cohort (Fig. 8C). The verification results of the expression patterns of prognostic genes in PRAD cell lines showed that the expressions of the 7 prognostic genes exhibited significant heterogeneity among different PRAD cell lines, and the cell lines with high expression of each gene were distinctly different. Among them, ABHD11 had the highest expression in H660 (Fig. 9A), CAT had the highest expression in LNCaP-abl (Fig. 9B), GSTZ1 had the highest expression in H660 (Fig. 9C), NTHL1 had the highest expression in H660 (Fig. 9D), OXR1 had the highest expression in H660 (Fig. 9E), PTRH2 had the highest expression in C4-2 (Fig. 9F), and SLC25A39 had the highest expression in PC3 (Fig. 9G).

## Discussion

Compared with other malignant tumors of the urinary system, the prognosis of PRAD is comparatively favorable, and the progression is slow [48]. Nevertheless, approximately 30% of patients with intermediate-to high-risk localized and locally advanced PRAD still experience recurrence or metastasis despite undergoing surgery and radiotherapy [49, 50]. The significance of TAMs in promoting tumor initiation and progression is increasingly acknowledged [51]. For example, TAMs in hepatocellular carcinoma facilitate tumor progression by upregulating hepatocyte growth factor expression [52]. TAMs in endometrial cancer promote tumor progression by secreting CXCL8 to reduce the expression of ERα [53]. In breast cancer and pancreatic ductal cancer, macrophage polarization is associated with poor prognosis of patients [54, 55]. Mitochondria, a crucial organelle, play a significant regulatory role during tumor progression by influencing the polarization of TAMs [13, 56].

In this study, prognostic genes related to mitochondria and macrophage polarization in PRAD were obtained through bioinformatics methods such as differential expression analysis, WGCNA, and MR: ABHD11, PTRH2, CAT, NTHL1, SLC25A39, OXR1, and GSTZ1. A prognostic model was constructed, and independent prognostic factors were obtained through independent prognostic tests. In addition, analyses such as GSEA, immune microenvironment, immunotherapy, and immune subtypes were conducted on high- and low-risk groups to elucidate the molecular mechanism of PRAD and provide a basis for the clinical treatment of PRAD.

αβ-Hydrolase domain-containing 11 (ABHD11) is a mitochondrial hydrolase associated with the 2-oxoglutarate (2-OG) dehydrogenase complex. It facilitates the conversion of 2-OG to succinyl-CoA by maintaining functional lipoylation [57]. Mechanistically, ABHD11 deficiency impairs the lipoylation process of this complex, thereby blocking 2-OG metabolism and leading to its accumulation [57]. This excess 2-OG inhibits α-ketoglutarate-dependent demethylases (such as JMJD3) in macrophages via epigenetic modifications. Consequently, it suppresses the expression of M1 polarization markers (e.g., TNF-α) while upregulating the expression of M2-associated genes (e.g., IL-10) [58, 59]. Simultaneously, accumulated 2-OG disrupts mitochondrial complex I activity, increasing reactive oxygen species (ROS) generation [60]. In TAMs, ROS activates STAT3 phosphorylation, further reinforcing the M2 polarization process [61]. Thus, ABHD11 likely regulates the mitochondrial-metabolic-immune axis in PRAD by modulating 2-OG levels, linking mitochondrial dysfunction to the remodeling of an immunosuppressive microenvironment.

Peptidyl-tRNA Hydrolase 2 (PTRH2) maintains mitochondrial integrity by stabilizing the key anti-apoptotic protein Bcl2, which effectively prevents mitochondrial outer membrane permeabilization [62]. At the molecular level, upregulation of PTRH2 in PRAD enhances the Bcl2-mediated suppression of mitochondrial outer membrane permeabilization, thereby reducing mitochondrial release of cytochrome c and ROS [63] This mechanism limits the release of damage-associated molecular patterns (DAMPs) linked to mitochondrial damage, such as mitochondrial DNA (mtDNA), which would otherwise activate the TLR9 receptor in macrophages, thereby promoting M1 polarization [64]. Concurrently, PTRH2 activates the FAK-Zeb1 signaling pathway in PRAD cells, promoting the secretion of IL-6 [64]. Following its binding to receptors on TAMs, IL-6 induces the production of M2 markers (e.g., CCL22) via STAT3 activation. Thus, PTRH2 links mitochondrial survival signaling to macrophage polarization processes by reducing levels of pro-inflammatory DAMPs and enhancing pro-tumorigenic cytokine secretion.

Catalase (CAT), a peroxisomal/mitochondrial enzyme responsible for degrading H₂O₂, exhibits reduced expression levels in PRAD tissues, leading to the accumulation of ROS [65]. This ROS surge triggers two interdependent effects along the mitochondria-macrophage axis: (1) Mitochondrial ROS oxidize mitochondrial DNA, releasing DAMPs that activate the cGAS-STING pathway in TAMs. This promotes M2 polarization via interferon-β-dependent PD-L1 upregulation [66]; (2) Elevated H₂O₂ stabilizes HIF-1α in macrophages, shifting their metabolism towards glycolysis and fatty acid oxidation—hallmark metabolic features of M2-polarized TAMs [67, 68]. Collectively, this mechanism establishes a feedforward loop: mitochondrial oxidative stress initiated by CAT deficiency subsequently enhances the immunosuppressive function of macrophages, thereby accelerating PRAD progression.

Endonuclease III-like protein 1 (NTHL1) is a base excision repair glycosylase that maintains mtDNA integrity by repairing oxidative base damage [69]. In PRAD models, upregulation of NTHL1 enhances mtDNA repair capacity, reducing mtDNA strand breaks and the release of DAMPs, such as 8-hydroxyguanine [70]. At the molecular level, decreased levels of DAMPs derived from mtDNA inhibit Toll-like receptor 4 (TLR4) activation in macrophages. Consequently, this suppresses NF-κB-mediated M1 polarization, evidenced by reduced interleukin-1 beta (IL-1β) expression [71]. Furthermore, NTHL1-mediated mtDNA stability sustains the activity of mitochondrial complex IV, thereby lowering ROS generation [70]. The resulting lower ROS levels in PRAD cells diminish H₂O₂-induced transforming growth factor beta (TGF-β) secretion. Thus, NTHL1 links mitochondrial genome stability to macrophage polarization by suppressing pro-inflammatory signaling [71].

SLC25A39 is a mitochondrial carrier protein responsible for importing glutathione (GSH) into the mitochondria, thereby protecting against oxidative stress [72]. In PRAD cells, upregulation of SLC25A39 elevates intramitochondrial GSH levels, reducing mitochondrial reactive oxygen species (mtROS) by enhancing superoxide anion scavenging [73]. At the cellular level, lower mtROS levels decrease CCL2 secretion by PRAD cells. This chemokine recruits monocytes and promotes their polarization into M2-type TAMs [74]. At the molecular level, SLC2539-mediated GSH transport maintains mitochondrial fatty acid oxidation by preserving aconitase activity [72]. This process increases succinate secretion by PRAD cells, which subsequently activates the GPR91 receptor on macrophages. This activation promotes IL-1β production and drives M1 polarization. However, this effect is counteracted by the reduced CCL2 levels, ultimately resulting in a net shift towards the M2 phenotype [75]. Collectively, these findings demonstrate that SLC25A39 regulates the mitochondrial-immune axis through redox and metabolic signaling pathways.

OXR1, an antioxidant gene localized to mitochondria, scavenges mtROS by activating superoxide dismutase 2 [76]. In PRAD cells, downregulation of OXR1 expression reduces superoxide dismutase 2 activity, leading to elevated mtROS levels and triggering the opening of the mitochondrial permeability transition pore [77]. This results in the release of mtDNA into the cytosol, which activates the NLRP3 inflammasome within PRAD cells and subsequently promotes IL-1β secretion [78]. IL-1β binds to the IL-1 receptor on the macrophage surface, activating the MAPK signaling pathway and upregulating expression of M1 markers (e.g., CD86). Additional studies indicate that sustained mtROS also induces PRAD cells to secrete prostaglandin E2. prostaglandin E2 suppresses NLRP3 protein in macrophages and promotes their polarization toward the M2 phenotype [78]. Consequently, OXR1 deficiency establishes a dual signaling effect: initially promoting M1 polarization, while ultimately stabilizing the M2 polarization state. This mechanism links mitochondrial redox homeostasis to dynamic immune modulation.

GSTZ1, a glutathione S-transferase, exhibits context-dependent functions across various cancers, closely associated with tissue-specific microenvironments. In hepatocellular carcinoma, GSTZ1 exerts tumor-suppressive effects: its downregulation leads to succinylacetone accumulation, activating the NRF2 signaling pathway and thereby promoting hepatocellular carcinoma progression [79, 80]. Conversely, in lung squamous cell carcinoma, elevated GSTZ1 expression supports tumor cell survival by enhancing the detoxification of reactive aldehydes [81].

In PRAD, the unique microenvironment characterized by androgen signaling, high macrophage infiltration, and mitochondrial metabolic reprogramming shapes the function of GSTZ1. Our data demonstrate upregulated GSTZ1 expression in PRAD (Fig. 8C). Mechanistic analyses reveal two specific roles of GSTZ1 in PRAD. First, mitochondrial succinylacetone metabolism: GSTZ1 metabolizes succinylacetone in PRAD cell mitochondria, preventing its inhibition of 2-OG dehydrogenase [80]. This maintains 2-OG metabolism and limits M2 macrophage polarization – an effect opposite to that of ABHD11 deficiency [57, 58]. Second, suppression of adenosine secretion: GSTZ1 upregulation inhibits adenosine secretion (a byproduct of mitochondrial ATP hydrolysis) by PRAD cells [81]. Within TAMs, adenosine acts as a critical M2 polarization signal by activating the A2A receptor [9]. By reducing adenosine levels, GSTZ1 indirectly suppresses TAM-mediated immunosuppression. This effect significantly differs from its roles in hepatocellular carcinoma or lung squamous cell carcinoma, where adenosine signaling plays a relatively minor role in the tumor microenvironment. Therefore, the upregulation of GSTZ1 in PRAD reflects an adaptive response to the tissue-specific microenvironment, balancing mitochondrial metabolism and immunological crosstalk to promote PRAD progression.

Analysis of immune microenvironment heterogeneity revealed distinct infiltration patterns between high- and low-risk PRAD groups, with activated CD4 + T cells, mast cells, Tregs, and immature dendritic cells (DCs) showing significant correlations with prognostic genes. Mast cells and neutrophils were found to impede PRAD progression through cytokine-mediated immune activation [82, 83]. Intriguingly, Tregs exhibited dual regulatory roles: while tumor tissues displayed enrichment of TH1, TH17, and Treg populations, Tregs promoted a pro-tumor microenvironment by suppressing NK cells and cytotoxic lymphocytes while fostering myeloid-derived suppressor cells, M2 macrophages, and cancer-associated fibroblasts [84, 85]. Notably, M2 macrophages demonstrated a direct positive correlation with disease progression, and preclinical studies targeting colony-stimulating factor-1 (CSF-1) to inhibit M2 polarization enhanced radiosensitivity [86, 87]. Within the tumor microenvironment, DCs undergo maturation upon uptake of tumor-derived molecules, including damage-associated molecular patterns such as heat shock proteins and high-mobility group box 1, along with proinflammatory cytokines, and subsequently migrate to T cell-rich secondary lymphoid organs. There, they activate cytotoxic CD8 + T cells and helper CD4 + T cells through MHC-antigen presentation and co-stimulatory signaling, ultimately orchestrating tumor cell elimination [88, 89].

Tumors can be classified into six immune subtypes based on the characteristics of the immune microenvironment: C1, C2, C3, C4, immune silent (C5), and TGF-β dominant (C6) [90]. Our findings indicate that there is no difference in the distribution of high-risk and low-risk groups between C1 and C4. However, among the other immune subtypes, there are significant differences in the distribution of high and low-risk groups. C2 is marked by high expression of IFN-γ and related immune activation genes and is commonly found in immunologically active tumors [90]. IFN-γ can activate CD8 + T cells and NK cells and enhance anti-tumor immunity, but a long-term high IFN-γ environment may lead to T cell exhaustion (such as upregulation of PD-1/PD-L1), resulting in immune escape [91]. For example, the IFN-γ high-expression subtype in lung cancer is sensitive to PD-1 inhibitors, but some patients relapse due to T cell exhaustion [92]. IFN-γ enhances antigen presentation by inducing MHC-I expression in tumor cells, but it may also select drug-resistant clones through immune editing and accelerate tumor evolution [93, 94]. C3 is dominated by infiltration of neutrophils and pro-inflammatory cytokines (such as IL-6, TNF-α) and is commonly found in tumors with HPV-related cancers or a background of chronic inflammation [95]. Tumor-associated neutrophils in the inflammatory tumor microenvironment promote invasion and metastasis by releasing ROS and matrix metalloproteinases [95]. For example, HPV + tumors in penile cancer recruit CD15 + myeloid cells through CXCL-8, forming an immunosuppressive microenvironment, which is associated with a poor prognosis [94, 95].

During the investigation of significantly enriched biological pathways between high-risk and low-risk groups, we observed marked differences in DNA damage repair (DDR) pathways. DNA repair constitutes a complex process that detects and rectifies various types of DNA damage through mechanisms including BER, nucleotide excision repair (NER), MMR, HRR, and non-homologous end joining (NHEJ) [96]. For instance, mutations in genes such as BRCA1/2 leading to HRR dysfunction are associated with metastatic castration-resistant PRAD (the PROfound trial confirmed) [97]. Patients with HRR deficiencies demonstrate sensitivity to PARP inhibitors (e.g., olaparib) based on the “synthetic lethality” principle [98]. MMR defects result in microsatellite instability (MSI), which correlates with high mutational burden in a subset of PRAD cases (approximately 3–5%) and may confer sensitivity to immune checkpoint inhibitors [99, 100]. BER and NER pathways participate in repairing chemotherapy/radiation-induced damage, where their activity levels might influence therapeutic outcomes [101, 102]. Concurrently, DDR defects drive PRAD progression. For example, loss of DDR functionality leads to mutation accumulation, activating oncogenic signaling pathways (e.g., PTEN deletion, TP53 mutations) that promote dedifferentiation and metastasis [103]. HRR-deficient tumors exhibit sensitivity to platinum-based chemotherapy but may show complex responses to conventional radiotherapy due to differential repair capacities [104, 105]. Upregulation of BER/NER pathways might diminish the efficacy of alkylating agents (e.g., temozolomide) or radiotherapy [106]. DDR deficiencies accelerate clonal evolution, potentially fostering resistant subpopulations (e.g., Androgen receptor signaling variants or neuroendocrine differentiation) [106–108].

This study conducted a drug sensitivity analysis on different risk groups of PRAD. The results showed good sensitivity to bexarotene, CCT018159, FH535, lapatinib, and MG.132. Bexarotene, as a synthetic retinoid analogue, can bind and activate RXR, playing a crucial role in regulating the life activities of tumor cells [109]. Moreover, its combination with chemotherapy drugs is effective for lung cancer, etc., and it can also reduce the chemotherapy resistance rate of PC3 cells [110]; CCT018159 is a heat shock protein inhibitor, which can arrest the tumor cell cycle or induce apoptosis [111, 112]; FH535, as a β-catenin inhibitor, can inhibit the growth of DU145 cells [113]; Lapatinib can block the ErbB2 signaling pathway, and its combination with abiraterone can enhance the therapeutic effect [114, 115]; MG-132 can sensitize PRAD cells that are resistant to TRAIL [116]. Therefore, the results of drug sensitivity analysis provide new theoretical support for the clinical drug treatment of PRAD.

We identified 7 prognostic genes related to macrophage polarization and mitochondria in PRAD (ABHD11, PTRH2, CAT, NTHL1, SLC25A39, OXR1, and GSTZ1), through a series of bioinformatics methods, and explored the independent prognostic factors, immune defense mechanisms, and chemotherapeutic drug sensitivity of PRAD, providing a new theoretical basis for exploring the immune defense mechanisms and targeted therapeutic drugs in PRAD.

However, this study has several limitations. Firstly, the clinical cohort comprising all PRAD cases was derived solely from public databases, lacking multi-center cohorts. This may limit the clinical generalizability of the findings. Secondly, due to the unavailability of suitable cancer cell line datasets, we were unable to validate the concordance between gene expression profiles in clinical samples and the bioinformatic predictions regarding prognostic genes. More critically, all conclusions are based on bioinformatic predictions and lack in vitro/in vivo experimental validation of the functional roles and underlying mechanisms of the seven prognostic genes. For instance, the postulated regulatory roles of ABHD11 and SLC25A39 in macrophage polarization have not been experimentally validated.

To address these limitations, future research will integrate multi-center clinical resources to establish a prostate cancer sample bank encompassing different pathological stages and treatment regimens. The clinical application value of the risk model will be validated through retrospective cohort studies. Meanwhile, efforts will continue to identify suitable publicly available datasets to verify gene expression consistency. Prioritize functional validation experiments, including gene editing experiments based on the THP-1 macrophage model (to detect polarization markers), co-culture experiments with PRAD cell lines, mitochondrial function analysis, and xenograft experiments in nude mice, aiming to elucidate the functional mechanisms of the relevant genes.

## Conclusions

This study employed bioinformatic analyses utilizing the TCGA-PRAD dataset, a mitochondrial-related gene set, and a macrophage polarization-related gene set to identify 103 candidate genes. Subsequently, a prognostic model was constructed via multivariate regression analysis, ultimately identifying 7 prognostic genes exhibiting a significant positive association with PRAD progression. Functional and immune analyses demonstrated that these genes may cooperatively regulate the “mitochondrial metabolism - macrophage polarization” axis, providing a novel mechanistic framework for understanding the interplay between mitochondrial dysfunction and immune microenvironment remodeling in PRAD. Assessment of immunotherapy response and drug sensitivity indicated that high-risk patients with elevated TMB and distinct immune checkpoint expression profiles may benefit from combination therapy involving Bexarotene or CCT018159. Future research should validate the roles of these genes in regulating mitochondrial respiration and macrophage polarization, explore their clinical utility in guiding neoadjuvant immunotherapy, and investigate whether targeting the “ABHD11/SLC25A39 - mitochondrial metabolites - M2 macrophages” axis can reverse therapeutic resistance in advanced PRAD patients.

In summary, our findings not only reveal novel prognostic biomarkers but also propose a potential therapeutic target axis, laying the groundwork for precision medicine in PRAD.

## Supplementary Information

Below is the link to the electronic supplementary material.

Supplementary Material 1.

## Data Availability

The datasets analyzed during the current study are available in public, open-access repositories listed in this article. The datasets we analyzed during the current study are available in the GEO datasets(https://www.ncbi.nlm.nih.gov/geo/): GSE70769; TCGA-PRAD datasets (https://xenabrowser.net/datapages/, accessed on 25 September 2024); MitoCarta 3.0 datasets (https://personal.brPRADdinstitute.org/scalvo/MitoCarta3.0/).; Molecular Signatures Database (https://www.gsea-msigdb.org/gsea/msigdb/), the search keyword is “Macrophage Polarization”; IEU OpenGWAS datasets(https://gwas.mrcieu.ac.uk/).

## Abbreviations

PRAD: Prostate adenocarcinoma
TAMs: Tumor-associated macrophages
ROS: Reactive oxygen species
MR: Mendelian randomization
SNP: Single-nucleotide polymorphism
TCGA-PRAD: The Cancer Genome Atlas prostate adenocarcinoma
GEO: Gene Expression Omnibus
MRGs: Mitochondria-related genes
MPRGs: Macrophage polarization-related genes
CGs: Candidate genes
DEGs: Differentially expressed genes
GWAS: The Genome-Wide Association Studies
eQTL: Expression Quantitative Trait Loci
FC: Fold Change
WGCNA: Weighted gene co-expression network analysis
BCR-FS: Biochemical recurrence-free survival
KM: The Kaplan-Meier
GO: Gene Ontology
KEGG: Kyoto Encyclopedia of Genes and Genomes
MF: Molecular function
CC: Cellular component
BP: Biological process
LD: Linkage Disequilibrium
IVW: Inverse variance weighted
OR: Odds ratio
LOO: Leave-One-Out
HR: Hazard ratio
PH: Proportional hazards
LASSO: Least absolute shrinkage and selection operator
HRC: High-risk cohort
LRC: Low risk cohort
ROC: The receiver operating characteristic
AUC: The area under the curve
NES: Normalized enrichment score
GSEA: The gene set enrichment analysis
DC: Dendritic cells
TIDE: Tumor immune dysfunction and exclusion
TMB: Tumor mutation burden
IPS: Immunophenoscore
IC50: 50% inhibiting concentration
DDR: DNA damage repair
BER: Base excision repair
NER: Nucleotide excision repair
MMR: Mismatch repair
HRR: Homologous recombination repair
GSH: Glutathione
NHEJ: Non-homologous end joining
mtROS: Mitochondrial reactive oxygen species
2-OG: 2-oxoglutarate
