# Supplementary material for: Identification of prognostic genes associated with mitochondria and macrophage polarization in prostate adenocarcinoma based on transcriptome and Mendelian randomization analysis
Source: Discov Oncol. 2025 Dec 31;17:5. doi: 10.1007/s12672-025-03858-5 (PMC12770125; doi:10.1007/s12672-025-03858-5)
Supplement: Supplementary file 1 — Supplementary Material 1. [file 12672_2025_3858_MOESM1_ESM.zip › 12672_2025_3858_MOESM1_ESM/STROBE-MR-checklist.docx]

**STROBE-MR checklist of recommended items to address in reports of Mendelian randomization studies**^1^ ^2^

| **Item No.** | **Section** | **Checklist item** | **Page No.** | **Relevant text from manuscript** |
| --- | --- | --- | --- | --- |
| 1 | **TITLE and ABSTRACT** | Indicate Mendelian randomization (MR) as the study’s design in the title and/or the abstract if that is a main purpose of the study | 1 | Identification of prognostic genes associated with mitochondria and macrophage polarization in prostate adenocarcinoma based on transcriptome and Mendelian randomization analysis |
|  | **INTRODUCTION** |  |  |  |
| 2 | **Background** | Explain the scientific background and rationale for the reported study. What is the exposure? Is a potential causal relationship between exposure and outcome plausible? Justify why MR is a helpful method to address the study question | 3 | Introduction: paragraphs 4  Mendelian randomization (MR) analysis uses genetic variants, such as single-nucleotide polymorphisms (SNPs), that… offers a new theoretical basis for researching and treating PRAD. |
| 3 | **Objectives** | State specific objectives clearly, including pre-specified causal hypotheses (if any). State that MR is a method that, under specific assumptions, intends to estimate causal effects | 3 | Introduction: paragraphs 5  This study seeks to identify prognostic genes associated with mitochondria and macrophage polarization in PRAD by integrating transcriptome data with MR analysis, thereby offering novel therapeutic targets for the disease. Based on these prognostic genes, a predictive model is developed to examine the biological pathways involved in high- and low-risk patient groups, and to investigate differences in their immune mi-croenvironment, immunotherapy response, and drug sensitivity. This offers a new theoretical basis for researching and treating PRAD. |
|  | **METHODS** |  |  |  |
| 4 | **Study design and data sources** | Present key elements of the study design early in the article. Consider including a table listing sources of data for all phases of the study. For each data source contributing to the analysis, describe the following: |  |  |
|  | a) | Setting: Describe the study design and the underlying population, if possible. Describe the setting, locations, and relevant dates, including periods of recruitment, exposure, follow-up, and data collection, when available. | 3、5 | 1. 2.1 Data collection  The Genome-Wide Association Studies (GWAS) data of expression Quantitative Trait Loci (eQTL) of candidate genes (CGs) were searched from the Integrative Epidemiolo-gy Unit (IEU) open GWAS (https://gwas.mrcieu.ac.uk/). The ''prostate cancer'' was a keyword from the IEU open GWAS database to obtain the ukb-b-13348 dataset. There were 9,851,867 SNPs from 462,933 samples (case: 3,269, control: 459,664). The popula-tion was European.  2. 2.5 Mendelian randomization analysis |
|  | b) | Participants: Give the eligibility criteria, and the sources and methods of selection of participants. Report the sample size, and whether any power or sample size calculations were carried out prior to the main analysis | 5 | 2.5 Mendelian randomization analysis |
|  | c) | Describe measurement, quality control and selection of genetic variants | 5 | 2.5 Mendelian randomization analysis. |
|  | d) | For each exposure, outcome, and other relevant variables, describe methods of assessment and diagnostic criteria for diseases | 5 | 2.5 Mendelian randomization analysis. |
|  | e) | Provide details of ethics committee approval and participant informed consent, if relevant |  | N/A |
| 5 | **Assumptions** | Explicitly state the three core IV assumptions for the main analysis (relevance, independence and exclusion restriction) as well assumptions for any additional or sensitivity analysis | 5 | 2.5 Mendelian randomization analysis. |
| 6 | **Statistical methods: main analysis** | Describe statistical methods and statistics used |  |  |
|  | a) | Describe how quantitative variables were handled in the analyses (i.e., scale, units, model) |  | This study was conducted leveraging data from previous publication or public databank. We applied no particular treatment in analyses concerning GWAS data of quantitative variables |
|  | b) | Describe how genetic variants were handled in the analyses and, if applicable, how their weights were selected | 5 | 2.5 Mendelian randomization analysis |
|  | c) | Describe the MR estimator (e.g. two-stage least squares, Wald ratio) and related statistics. Detail the included covariates and, in case of two-sample MR, whether the same covariate set was used for adjustment in the two samples | 5 | 2.5 Mendelian randomization analysis. |
|  | d) | Explain how missing data were addressed |  | No missing data need to be addressed in this study |
|  | e) | If applicable, indicate how multiple testing was addressed | 5 | 2.5 Mendelian randomization analysis. |
| 7 | **Assessment of assumptions** | Describe any methods or prior knowledge used to assess the assumptions or justify their validity | 5 | 2.5 Mendelian randomization analysis. |
| 8 | **Sensitivity analyses and additional analyses** | Describe any sensitivity analyses or additional analyses performed (e.g. comparison of effect estimates from different approaches, independent replication, bias analytic techniques, validation of instruments, simulations) | 5 | 2.5 Mendelian randomization analysis. |
| 9 | **Software and pre-registration** |  |  |  |
|  | a) | Name statistical software and package(s), including version and settings used | 8 | Data were analyzed using R software (version 4.2.2) and GraphPad Prism version 8.0. The Wilcoxon rank-sum test was employed for comparisons, with statistical significance set at p < 0.05. |
|  | b) | State whether the study protocol and details were pre-registered (as well as when and where) |  | N/A |
|  | **RESULTS** |  |  |  |
| 10 | **Descriptive data** |  |  |  |
|  | a) | Report the numbers of individuals at each stage of included studies and reasons for exclusion. Consider use of a flow diagram | 11 | 3.3. MR analysis  Supplementary Table 5 |
|  | b) | Report summary statistics for phenotypic exposure(s), outcome(s), and other relevant variables (e.g. means, SDs, proportions) | 11 | 3.3. MR analysis |
|  | c) | If the data sources include meta-analyses of previous studies, provide the assessments of heterogeneity across these studies |  | N/A |
|  | d) | For two-sample MR:  i.  Provide justification of the similarity of the genetic variant-exposure associations between the exposure and outcome samples  ii.  Provide information on the number of individuals who overlap between the exposure and outcome studies |  | N/A |
| 11 | **Main results** |  |  |  |
|  | a) | Report the associations between genetic variant and exposure, and between genetic variant and outcome, preferably on an interpretable scale | 11 | 3.3. MR analysis  Supplementary Table 5 |
|  | b) | Report MR estimates of the relationship between exposure and outcome, and the measures of uncertainty from the MR analysis, on an interpretable scale, such as odds ratio or relative risk per SD difference | 11 | 3.3. MR analysis |
|  | c) | If relevant, consider translating estimates of relative risk into absolute risk for a meaningful time period |  | N/A |
|  | d) | Consider plots to visualize results (e.g. forest plot, scatterplot of associations between genetic variants and outcome versus between genetic variants and exposure) |  | Supplementary Figure 1-4, |
| 12 | **Assessment of assumptions** |  |  |  |
|  | a) | Report the assessment of the validity of the assumptions | 11 | 3.3. MR analysis |
|  | b) | Report any additional statistics (e.g., assessments of heterogeneity across genetic variants, such as *I^2^*, Q statistic or E-value) | 11 | 3.3. MR analysis  Example: OXR1 gene Q=12.75, Q df = 31, Q pval = 0.998449 (no heterogeneity); CAT gene Q = 14.06, Qdf = 30, Q pval = 0.994051 (no heterogeneity). There was no horizontal pleiotropy between 13 genes and PRAD (p > 0.05) (Supplementary Table 5). Example: OXR1 gene egger intercept = -0.00026, se=0.000156, p = 0.105729; CAT gene egger intercept = -0.000125, se = 6.24E-05, p = 0.053641. |
| 13 | **Sensitivity analyses and additional analyses** |  |  |  |
|  | a) | Report any sensitivity analyses to assess the robustness of the main results to violations of the assumptions | 11 | Sensitivity analyses of the 13 genes and PRAD yielded 13 genes for no heterogeneity (p > 0.05) (Supplementary Table 4). |
|  | b) | Report results from other sensitivity analyses or additional analyses | 11 | Analyzing the correlation between exposure factors and outcomes in the scatter plot, 11 risk factors were positively correlated, while OXR1 and CAT genes were negatively correlated (Supplementary Figure 1). Forest plots demonstrated that the effect value of OXR1 and CAT genes was less than 0, the effect value of 11 risk factors was greater than 0 (Supplementary Figure 2). The single-nucleotide polymorphism (SNP) numbers were largely symmetrical on both sides of the IVW line and conformed to Mendel's second law (Supplementary Figure 3). Sensitivity analyses of the 13 genes and PRAD yielded 13 genes for no heterogeneity (p > 0.05) (Supplementary Table 4). Example: OXR1 gene Q=12.75, Q df = 31, Q pval = 0.998449 (no heterogeneity); CAT gene Q = 14.06, Qdf = 30, Q pval = 0.994051 (no heterogeneity). There was no horizontal pleiotropy between 13 genes and PRAD (p > 0.05) (Supplementary Table 5). |
|  | c) | Report any assessment of direction of causal relationship (e.g., bidirectional MR) | 11 | A two-sample MR analysis was performed with 103 CGs as exposure factors and PRAD as the outcome |
|  | d) | When relevant, report and compare with estimates from non-MR analyses |  | N/A |
|  | e) | Consider additional plots to visualize results (e.g., leave-one-out analyses) | 11 | In Leave-One-Out (LOO), there were no points of serious bias, and the reliability of the results was illustrated (Supplementary Figure 4). |
|  | **DISCUSSION** |  |  |  |
| 14 | **Key results** | Summarize key results with reference to study objectives | 23 | In our study, bioinformatics was used to obtain 103 candidate genes through the TCGA-PRAD dataset, the mitochondrial-related gene dataset, and the macrophage polarization-related gene dataset. By combining MR analysis and constructing a prognostic model, 7 prognostic genes that have a positive causal effect in the progression of PRAD were screened out. In addition, a preliminary assessment of the immune therapeutic response and chemotherapeutic drug sensitivity of PRAD patients with different risk groups was conducted. Finally, based on a series of functional studies, it was found that these 7 certain genes may play a role in disease progression of PRAD by affecting mitochondrial function and macrophage polarization, and affecting the survival of patients. This finding provides valuable insights into revealing the molecular mechanism of the disease. |
| 15 | **Limitations** | Discuss limitations of the study, taking into account the validity of the IV assumptions, other sources of potential bias, and imprecision. Discuss both direction and magnitude of any potential bias and any efforts to address them | 23 | First, the study had a limited sample size, and all PRAD cases' clinical cohorts came from publicly available databases. Larger clinical samples will be used in future research to increase the accuracy of the findings. Second, there is no in vitro experimen-tation to validate the findings of this study. |
| 16 | **Interpretation** |  |  |  |
|  | a) | Meaning: Give a cautious overall interpretation of results in the context of their limitations and in comparison with other studies | 19 | 4. Discussion: paragraphs 2  In this study, prognostic genes related to mitochondria and macrophage polarization in PRAD were obtained through bioinformatics methods such as differential expression analysis, WGCNA, and MR: ABHD11, PTRH2, CAT, NTHL1, SLC25A39, OXR1, and GSTZ1. A prognostic model was constructed, and independent prognostic factors were obtained through independent prognostic tests. In addition, analyses such as GSEA, immune microenvironment, immunotherapy, and immune subtypes were conducted on high- and low-risk groups to elucidate the molecular mechanism of PRAD and provide a basis for the clinical treatment of PRAD. |
|  | b) | Mechanism: Discuss underlying biological mechanisms that could drive a potential causal relationship between the investigated exposure and the outcome, and whether the gene-environment equivalence assumption is reasonable. Use causal language carefully, clarifying that IV estimates may provide causal effects only under certain assumptions | 19-23 | 4. Discussion: paragraphs 3-13 |
|  | c) | Clinical relevance: Discuss whether the results have clinical or public policy relevance, and to what extent they inform effect sizes of possible interventions | 19-23 | 4. Discussion: paragraphs 3-13 |
| 17 | **Generalizability** | Discuss the generalizability of the study results (a) to other populations, (b) across other exposure periods/timings, and (c) across other levels of exposure | 23 | 4. Discussion: paragraphs 14  This study identified 7 prognostic genes related to macrophage polarization and mitochondria in PRAD, namely PTRH2, SLC25A39, OXR1, ABHD11, NTHL1, CAT, and GSTZ1, … seven prognostic genes and their roles will be experimentally verified in the future. |
|  | **OTHER INFORMATION** |  |  |  |
| 18 | **Funding** | Describe sources of funding and the role of funders in the present study and, if applicable, sources of funding for the databases and original study or studies on which the present study is based | 31 | No funding was received. |
| 19 | **Data and data sharing** | Provide the data used to perform all analyses or report where and how the data can be accessed, and reference these sources in the article. Provide the statistical code needed to reproduce the results in the article, or report whether the code is publicly accessible and if so, where |  | All data generated or analyzed during the present study was downloaded from GEO da-tasets(https://www.ncbi.nlm.nih.gov/geo/): GSE70769; TCGA-PRAD dataset s(https://xenabrowser.net/datapages/, accessed on 25 September 2024); MitoCarta 3.0 da-tasets(https://personal.brPRADdinstitute.org/scalvo/MitoCarta3.0/).; Molecular Signatures Data-base (https://www.gsea-msigdb.org/gsea/msigdb/), the search keyword is "Macrophage Polariza-tion"; IEU OpenGWAS datasets(https://gwas.mrcieu.ac.uk/). |
| 20 | **Conflicts of Interest** | All authors should declare all potential conflicts of interest | 31 | The authors declare no potential conflicts of interest. |

This checklist is copyrighted by the Equator Network under the Creative Commons Attribution 3.0 Unported (CC BY 3.0) license.

1. Skrivankova VW, Richmond RC, Woolf BAR, Yarmolinsky J, Davies NM, Swanson SA, et al. Strengthening the Reporting of Observational Studies in Epidemiology using Mendelian Randomization (STROBE-MR) Statement. JAMA. 2021;under review.

2. Skrivankova VW, Richmond RC, Woolf BAR, Davies NM, Swanson SA, VanderWeele TJ, et al. Strengthening the Reporting of Observational Studies in Epidemiology using Mendelian Randomisation (STROBE-MR): Explanation and Elaboration. BMJ. 2021;375:n2233.
