# Supplementary material for: Identification of prognostic genes associated with mitochondria and macrophage polarization in prostate adenocarcinoma based on transcriptome and Mendelian randomization analysis
Source: Discov Oncol. 2025 Dec 31;17:5. doi: 10.1007/s12672-025-03858-5 (PMC12770125; doi:10.1007/s12672-025-03858-5)
Supplement: Supplementary file 1 — Supplementary Material 1. [file 12672_2025_3858_MOESM1_ESM.zip › 12672_2025_3858_MOESM1_ESM/Supplementary Figure.docx]

**
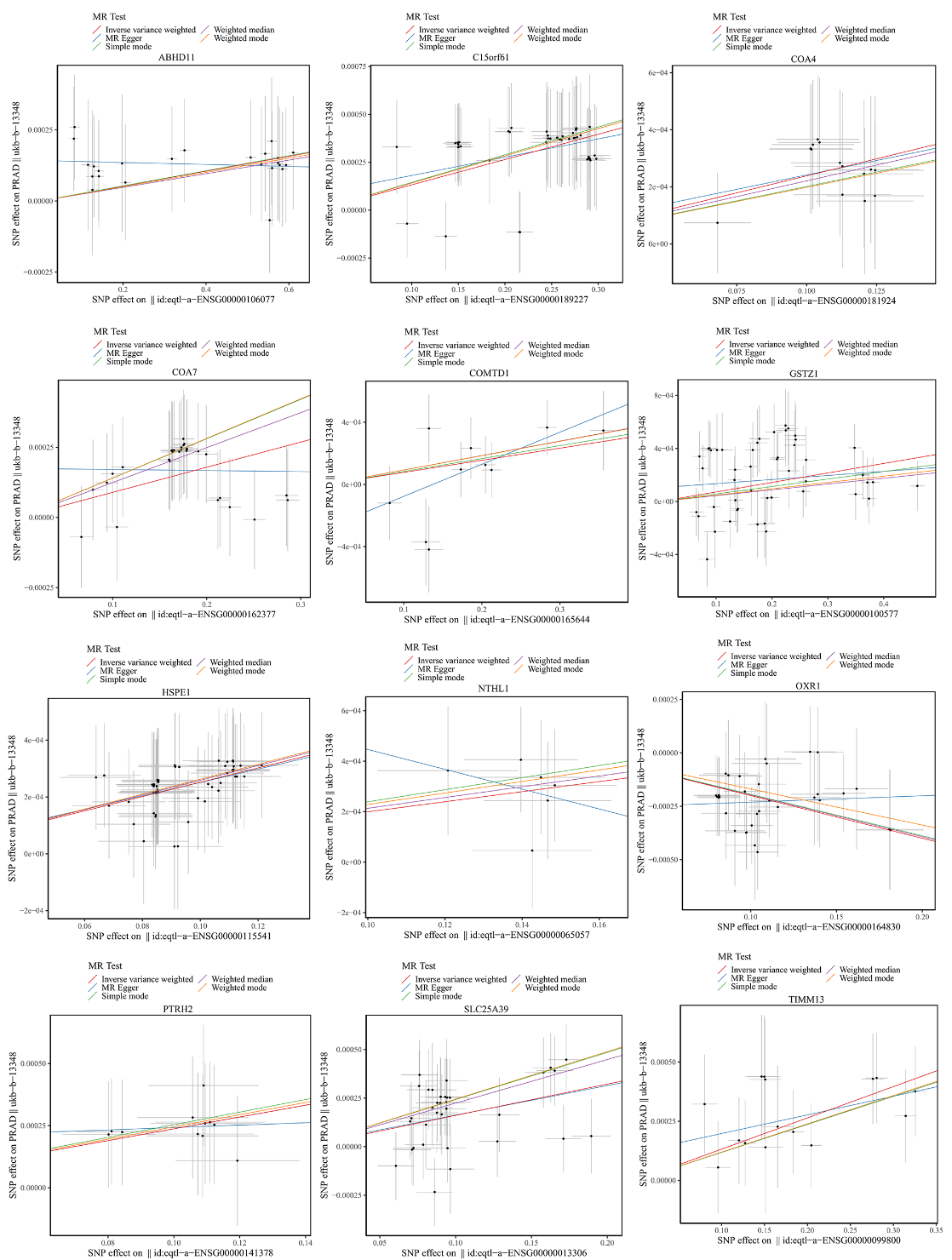
**

Supplementary Figure 1: Scatter plot showing the causal effect of 13 genes on the risk of PRAD. The horizontal coordinate is the effect of SNP on the exposure factor, and the vertical coordinate is the effect of SNP on the outcome. The colored lines represent the fitting results of different MR algorithms. A positive slope of the line indicates a risk factor, and a negative slope of the line indicates a safety factor. When the intercept is not 0, it implies that there may be confounding factors.


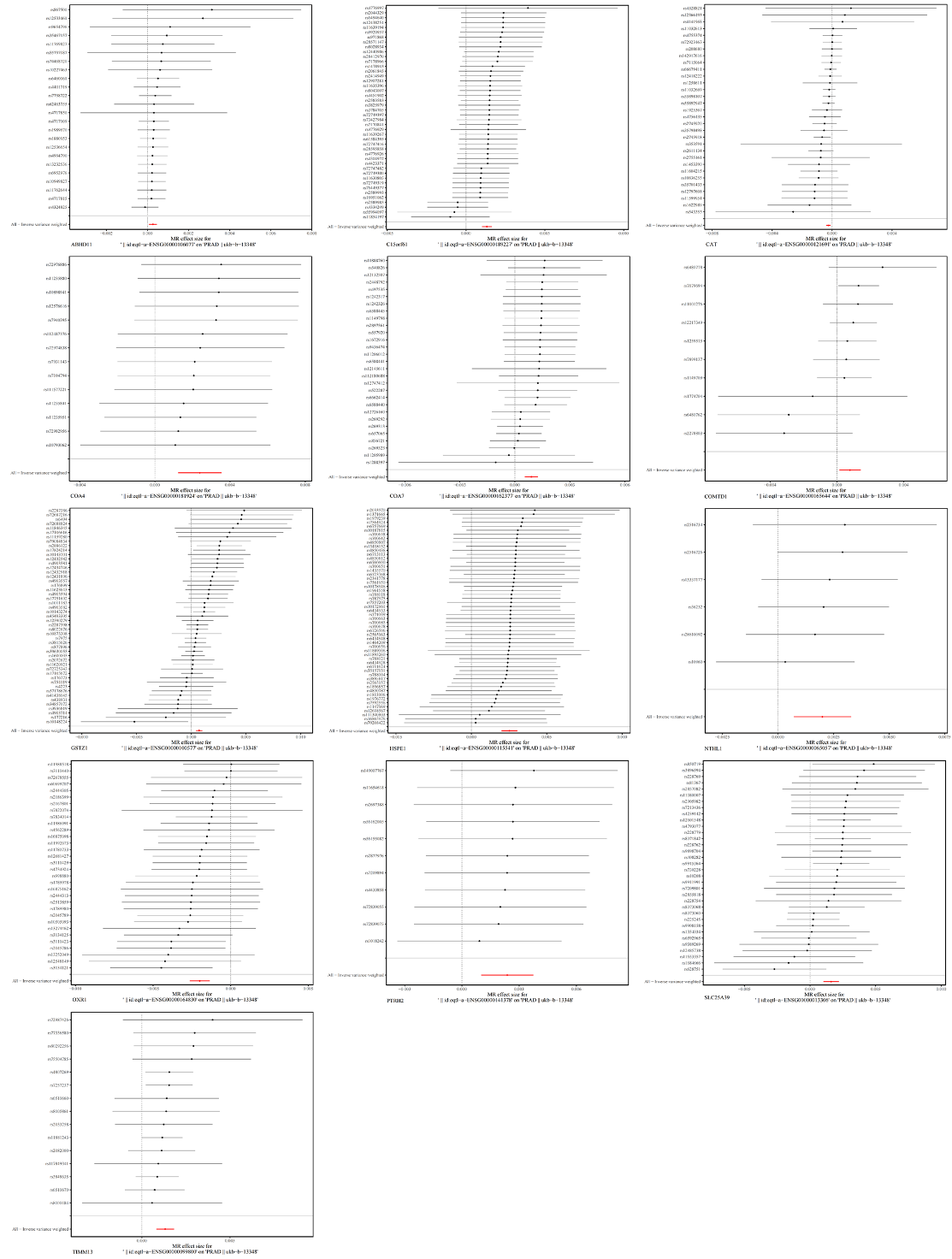


Supplementary Figure 2: Forest plot showing the causal effect of each 13 genes on the risk of PRAD. The horizontal axis is the effect value of each SNP locus on the outcome through the exposure factor, the vertical axis is the SNP locus, the red line is the over-all effect value, the black dot represents the IVW effect value after the SNP, and the red dot represents the IVW effect value of all SNPs.


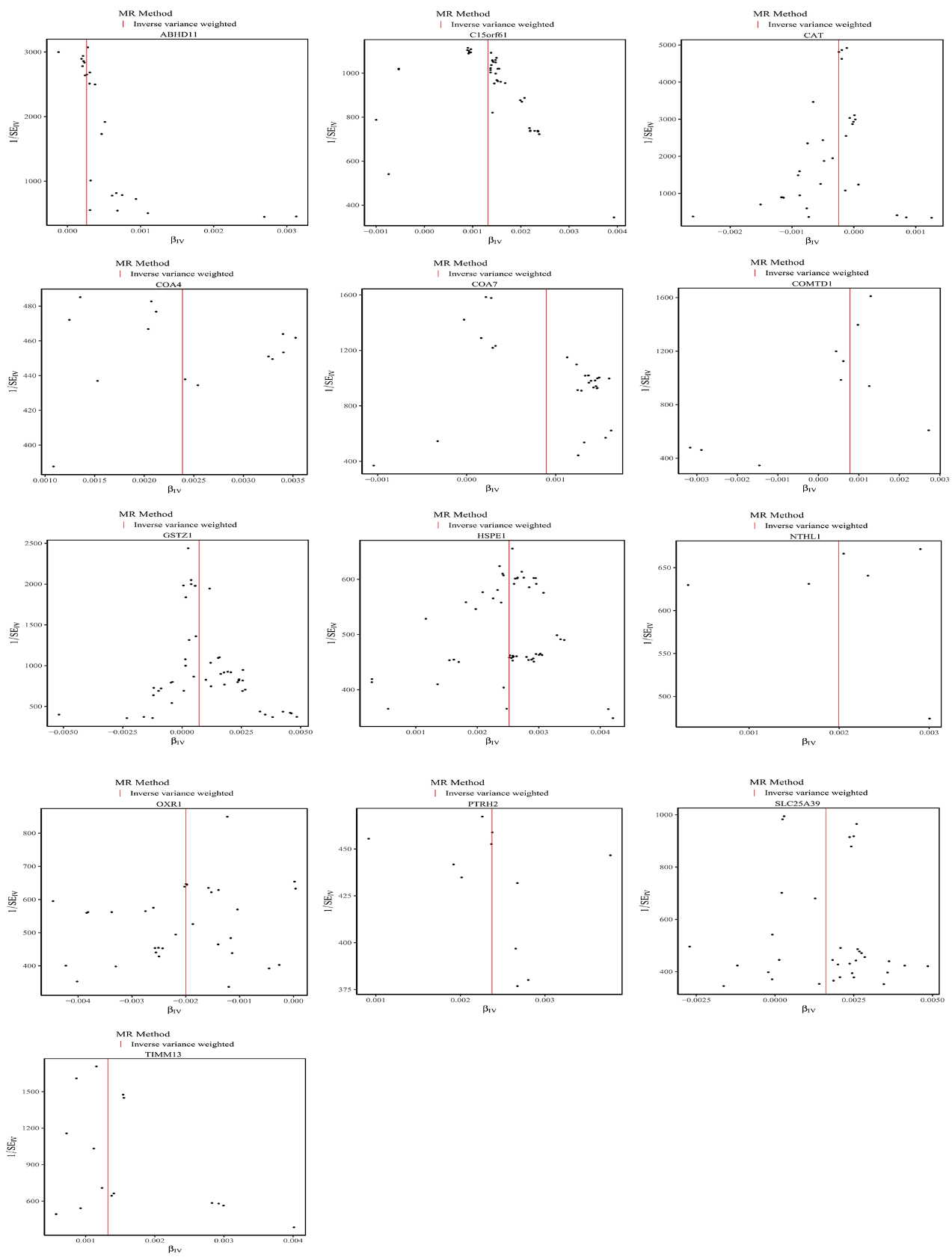


Supplementary Figure 3: Funnel plots to visualize the overall stochasticity of MR estimates for the effect of 13 genes on PRAD. The horizontal coordinate is the effect value of the instrumental variable, and the vertical coordinate is the reciprocal of the standard error.


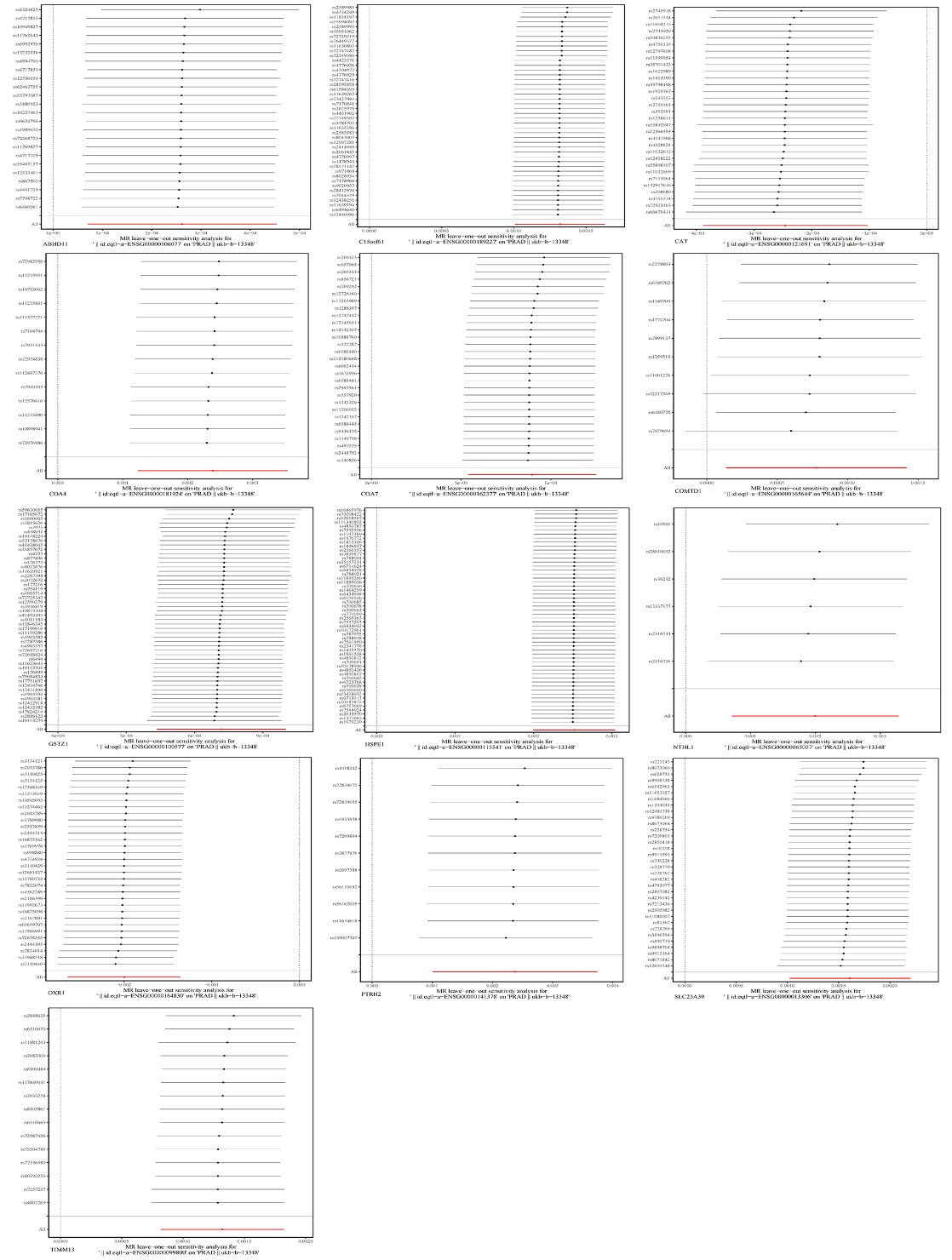


Supplementary Figure 4: Leave-one-out plot to visualize the causal effect of 13 genes on PRAD risk when leaving one SNP out. The horizontal axis is the effect value of each SNP locus on the outcome through the exposure factor, the vertical axis is the SNP locus, the red line is the overall effect value, the black dot represents the IVW estimate after removing the SNP, and the red dot represents the IVW estimate of all SNPs. If the horizontal deviation position of the black dot relative to the red dot is relatively close, it indicates that the MR result passes the test of the sensitivity analysis.


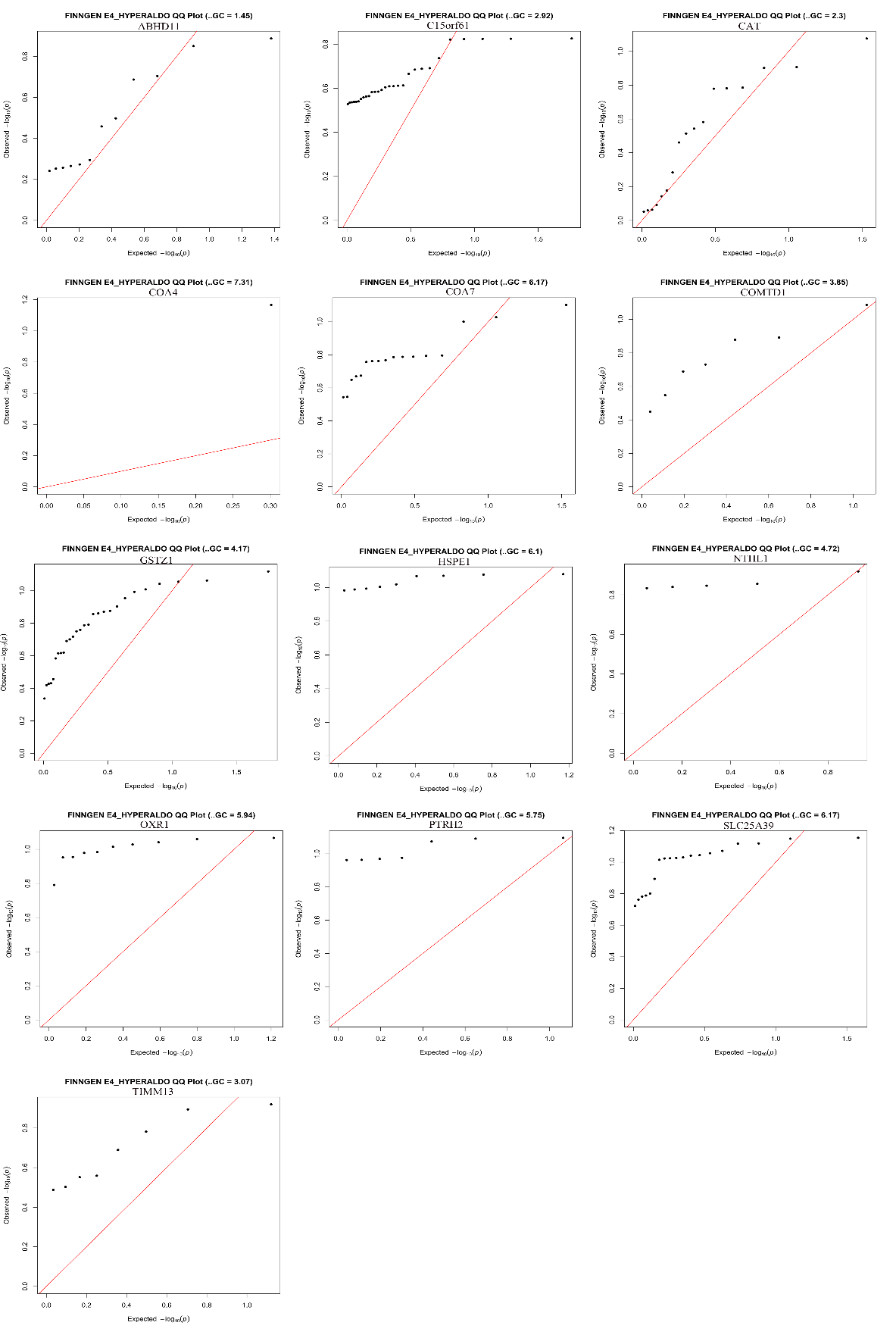


Supplementary Figure 5: Evaluate the gene inflation situation based on the λGC method. Use the λGC statistic to assess the p-value inflation effect in the association analysis of each of the 13 genes.
